# Assessment of urban microbiome assemblies with the help of targeted in silico gold standards

**DOI:** 10.1186/s13062-018-0225-6

**Published:** 2018-10-12

**Authors:** Samuel M. Gerner, Thomas Rattei, Alexandra B. Graf

**Affiliations:** 1 Department Bioengineering, University of Applied Sciences FH Campus Wien, Vienna, Austria; 2 Division of Computational System Biology, Department of Microbiology and Ecosystem Science, University of Vienna, Vienna, Austria

**Keywords:** Bioinformatics, Metagenome, Microbiome, Assembly, Binning, CAMDA challenge, In silico gold standard

## Abstract

**Background:**

Microbial communities play a crucial role in our environment and may influence human health tremendously. Despite being the place where human interaction is most abundant we still know little about the urban microbiome. This is highlighted by the large amount of unclassified DNA reads found in urban metagenome samples. The only in silico approach that allows us to find unknown species, is the assembly and classification of draft genomes from a metagenomic dataset. In this study we (1) investigate the applicability of an assembly and binning approach for urban metagenome datasets, and (2) develop a new method for the generation of in silico gold standards to better understand the specific challenges of such datasets and provide a guide in the selection of available software.

**Results:**

We applied combinations of three assembly (Megahit, SPAdes and MetaSPAdes) and three binning tools (MaxBin, MetaBAT and CONCOCT) to whole genome shotgun datasets from the CAMDA 2017 Challenge. Complex in silico gold standards with a simulated bacterial fraction were generated for representative samples of each surface type and city. Using these gold standards, we found the combination of SPAdes and MetaBAT to be optimal for urban metagenome datasets by providing the best trade-off between the number of high-quality genome draft bins (MIMAG standards) retrieved, the least amount of misassemblies and contamination. The assembled draft genomes included known species like *Propionibacterium acnes* but also novel species according to respective ANI values.

**Conclusions:**

In our work, we showed that, even for datasets with high diversity and low sequencing depth from urban environments, assembly and binning-based methods can provide high-quality genome drafts. Of vital importance to retrieve high-quality genome drafts is sequence depth but even more so a high proportion of the bacterial sequence fraction too achieve high coverage for bacterial genomes. In contrast to read-based methods relying on database knowledge, genome-centric methods as applied in this study can provide valuable information about unknown species and strains as well as functional contributions of single community members within a sample. Furthermore, we present a method for the generation of sample-specific highly complex in silico gold standards.

**Reviewers:**

This article was reviewed by Craig Herbold, Serghei Mangul and Yana Bromberg.

**Electronic supplementary material:**

The online version of this article (10.1186/s13062-018-0225-6) contains supplementary material, which is available to authorized users.

## Background

Microbes influence the way we live in ways far beyond anything we imagined just a decade ago. The results of the human microbiome project (HMP) showed an intricate interaction between the microbial communities within our body and our wellbeing [1, 2]. Even our mood is influenced by our microbiome [3, 4]. It is therefore no surprise that the research on the human microbiome has gained considerable momentum in the years following the HMP. In urban environments, millions of people interact with each other and the microbial communities that surround them (surface, air and water). The field of urban metagenomics analyses these communities and their influence on the wellbeing and health of citizens [5, 6]. For instance, studies have shown that the development and spread of antibiotics resistances is crucially influenced by the microbial environment [7, 8]. While some urban metagenome studies are already published [9, 10], more data and work is needed to be able to profile the metagenome of cities worldwide. To ensure reproducibility and transparency of urban metagenome research, the MetaSUB International Consortium [11] was formed.

Results from the first urban metagenome studies show that a large proportion of the DNA found in these samples is still not present in public databases and is therefore missing in any reference-based method. Additionally, urban microbiomes differ from other known microbiomes in their comparatively high population dynamics, especially considering areas where large numbers of people interact [9, 10].

To detect novel species and to enable a detailed analysis of microbe-microbe communities or host-microbe interactions (e.g., pathogenic and commensal), metagenomic reads have to be assembled into, ideally, complete genomes as read-to-database comparison methods would introduce biases. However, to our current knowledge, no other study tried to accomplish assemblies of urban microbiomes so far.

Many assemblers and genome binners, using a variety of approaches, are available for the purpose of metagenome assembly and classification. The computational performance and the quality of the resulting genome bins is in turn influenced by a multitude of sample and sequencing parameters. To help scientists dealing with this plethora of assembly tools, it is essential to provide clear assessment parameters and quality measures. The Critical Assessment of Metagenome Interpretation (CAMI) challenge provides a framework for developers to benchmark their programs on highly complex simulated datasets as well as an evaluation of present methods [12]. They reported very different assembly tool performance, depending on the features of the metagenome sample. These features include population diversity, sequencing quality and sequencing depth. High community diversity, especially the presence of closely related microbial strains, can decrease assembly performance dramatically and is one of the main challenges in metagenomics analysis. Recent benchmarks show that assemblers using multiple *k*-kmers for assembly substantially outperform single *k*-mer assemblers [12, 13]. Simulated data are essential for benchmarking as they are easily created for a multitude of experimental setups, but still represent only an approximation of reality and cannot replace a well-designed gold standard, as Mangul et al. [14] showed in their assessment of benchmarking approaches for omics tools.

To investigate the potential of de-novo assemblies and to detect unknown microorganisms in urban metagenome samples we performed metagenome assemblies and subsequent binning for the whole genome datasets of the cities Boston, Sacramento and New York provided in the CAMDA 2017 MetaSUB challenge [15]. The dataset consisted of 24 WGS samples from Boston and 18 metagenomic samples from Sacramento, as well as 24 randomly selected samples (of total 1572) from New York.

As the taxonomic composition of urban metagenome samples from CAMDA is unknown, we introduce sample-specific in silico gold standards to further assess tool performance with known-truths. Such ground truth is needed to not only compare results between samples of unknown origin but to assess recovery rates of genomes of known origin as well. Such benchmarking data sets mimic multiple parameters, such as microbial diversity on a large scale by using varying diversity of bacterial species, as well as varying diversity on a strain level of a single species [12]. These benchmarking sets consist purely of sampled or sequenced data from known genomes, creating synthetic communities which give full control on complexity of a community but do not replicate biological conditions of actual environmental samples.

A major difference of such benchmarking sets to real environmental data is the fraction of unknown sequences originating from yet unknown species, making it difficult to replicate similar conditions in synthetic data. Depending on sample origin, the fraction of unknown sequences can easily constitute half of all data as observed for urban metagenomes in New York [10]. To increase the representation of original conditions in our in silico gold standards, we took an alternative approach. We incorporate the unknown fraction of sequences in a sample while replacing all bacterial sequences we were able to classify with corresponding simulated sequences from reference genomes, creating a gold standard with a bacterial fraction of known-truths while still maintaining the original complexity of a sample as close as possible.

These in silico gold standards are then used to further assess assembly and binning performance specific to urban metagenomes.

## Methods

### Data description

The datasets were provided in the MetaSUB Challenge of the CAMDA 2017 [15] and we selected only WGS datasets from the three cities. The Boston dataset consisted of 24 samples with a sequencing depth between 0.2 Gbp and 11.8 Gbp per sample, the Sacramento dataset contained 18 samples with a sequencing depth between 5.1 Gbp and 6.4 Gbp per sample. The New York dataset consisted of 1572 samples, of which most were of low sequencing depth (1 Mbp to 19 Gbp with an average of 0.8 and a median of 0.6 Gbp). We randomly selected 24 samples from New York based on the distribution of the sequencing depth. Sequencing for all samples was done using an Illumina paired end protocol. The original read length was 101 bp for Boston, 126 bp for Sacramento, and 101–301 bp for New York. The Sacramento dataset consists of samples taken from benches (6), ticket machines (6) and platform railings (6) in stations, for the Boston dataset grips (6), poles (2), seats (5) and seat backs (2) in train cars, as well as touchscreens (9) at stations were swabbed. New York samples are only reported with surface type, with the selected samples originating from metal (14), wood (6) and metal/plastic (4) surfaces.

### Preprocessing

The sequence files were quality checked using FastQC version 0.11.5, and low quality reads were removed with Trimmomatic version 0.36 (filtering reads below a Phread-Score of 20 and a read length below 70 bp) [16]. Adapter sequences were removed by Cutadapt version 1.12 [17]. To filter all human reads, sequence files were aligned to the human reference genome hg38 by Bowtie2 version 2.3.0 [18] followed by extraction of all non-mapping reads by Samtools version 0.1.19 [19] and conversion back to FASTQ-Files using BEDtools bamtofastq version 2.21.0 [20].

### Assembly, binning and phylotyping

The tools were selected based on their performance in the CAMI challenge and additional benchmarking studies [12, 13, 21] as well as preliminary tests with a subset of samples. For all samples we compared the assemblies of MetaSPAdes and SPAdes version 3.11.1 [22, 23], and Megahit version v1.1.1–2-g02102e1 [24] in combination with three different binners, namely MaxBin version 2.2.2 [25], MetaBAT version 2.12.1 [26] and CONCOCT version 0.4.0 [27]. Assemblies were filtered for a minimum contig length of 500 bp, while binning was applied with default values (1000, 2500 and 1000 bp for minimum contig length for MaxBin, MetaBAT and CONCOCT respectively). All three binning methods use tetranucleotide frequencies and abundance information. Abundance information is obtained by helper scripts of CONCOCT and MetaBAT (Additional file 1), calculating the coverage per contig from mapped reads against their respective assembly as well as for pooled samples by mapping the single samples separately back to the assembly of the pool. Completeness and contamination of the resulting bins was analysed with CheckM version 1.0.7 [28] and phylogenomic inference of the bins was performed with AMPHORA version 2.0 [29].

The presence of 5S, 16S and 23S rRNA was predicted by barrnap version 0.9-dev [30] and tRNAs were predicted with tRNAscan-SE version 2.0 [31]. The presence of ribosomal clusters as well as tRNA content were both used as a criterion for high-quality genome drafts according to the MIMAG standards (Table 1) [32].

**Table 1:** MIMAG Standards

Quality level	Criteria
High-quality draft	Presence of 5S, 16S and 23S rRNAPresence of at least 18 tRNAs Completion > 90% Contamination < 5%
Medium-quality draft	Completion ≥50% Contamination < 10%
Low-quality draft	Completion < 50% Contamination < 10%

Selected set of criteria for low, medium and high-quality draft-genomes according to the Minimum Information about a Metagenome-Assembled Genome (MIMAG) standards [32]

To check the taxonomic identity of high-quality bins, genes were predicted with Prodigal [33] and the resulting Proteins were BLAST [34] searched against a local bacterial database (NCBI RefSeq - Jan. 2018). Average nucleotide identity (ANI) values were calculated with ANIcalculator [35] and average amino acid identity (AAI) values were calculated using a one to one BLAST search against the best Hit Organism from the previous search with an E-value cut-off of 0.05. Microbial phenotypes of high-quality genome drafts were predicted using the PICA framework [36] and PhenDB (https://phendb.csb.univie.ac.at/).

In silico bacterial replication measurements were performed using iRep version 1.1.14 [37]. iRep requires a minimum coverage of 5, less than 175 fragments/Mbp, less than 2% contamination and more than 75% completeness within the genome for calculation for a single genome draft bin. Additional mapping quality filters are applied during iRep calculation such as removing high- and low coverage windows and evaluation of coverage distribution by linear regression.

The resulting iRep value indicates the average proportion of respective species replicating in a sample of interest, such as an iRep value of 2 represents an average replication of every bacterium from the respective species or an iRep value of 1.34 an average replication of every third member in average.

### In silico gold standards

Sample-specific in silico gold standards, i.e. gold standards based on the taxonomic profile of a real sample, are created in a multi-step process. The first step is the taxonomic classification of all sequences within a sample to obtain read counts for single taxa from Centrifuge version 1.0.3-beta [38] with an index for prokaryotes, human and Viruses/Archaea (p + h + v). The p + h + v index is based on the NCBI RefSeq database (build on November 3rd, 2017). The p + h + v classification was used to extract all sequences classified as bacterial. To estimate the potentially unknown (unclassified) read content the Centrifuge nt index provided by the Centrifuge authors was used (index from June 12th, 2016).

In a second step, the output of Centrifuge is converted into a format used by Kraken [39] using the script centrifuge-kreport. All classified sequences on species or strain level are then matched to a reference genome in RefSeq. The exact number of reads classified by Centrifuge is sampled from the selected reference genomes using ART version 2.5.8. [40] applying matching error profiles, fragment and read lengths observed in the original sample.

In the last step, all sequences classified as bacterial are then removed from the original sample and replaced with the created in silico reads. The resulting in silico gold standard still constitutes only an approximation of the original sample, as classification of all bacterial sequences is dependent on databases, thereby not classifying all real bacterial sequences as such or to a close related species if the strain or species is not present in the database.

**Fig. 1:**
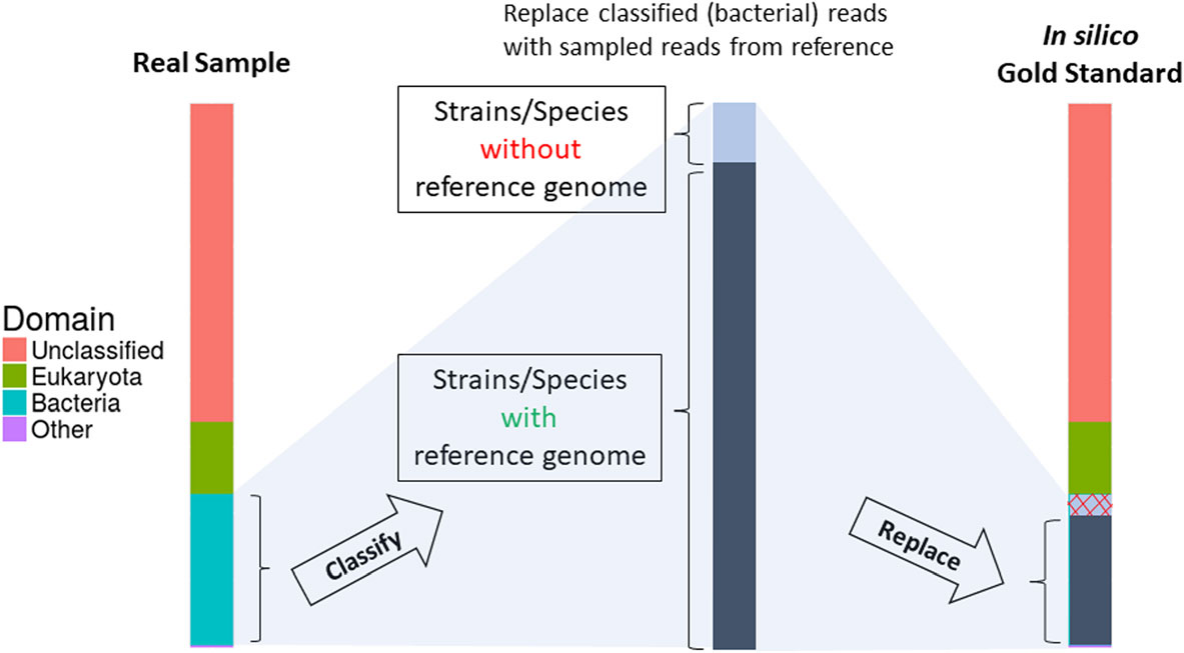
Composition of an in silico gold standard. The process of replacing classified bacterial reads with in silico reads obtained from known reference genomes is depicted. The resulting gold standard contains all unclassified sequences together with any non-bacterial sequences, while sequences classified as bacterial are replaced by in silico reads or, in case no reference genome could be assigned, are dropped

The whole workflow is schematically depicted in Additional file 2: Figure S1 together with the composition of an in silico gold standard created by the described approach (Fig. 1).

Assembly, binning and phylotyping is executed using the same approach as described for real samples. Assessment of misassemblies is achieved by MetaQUAST version 4.5 15ca3b9 [41] using high-quality genome drafts resulting from in silico gold standards compared to reference genomes sampled by at least 10,000 read pairs for the respective gold standard with matching phylotypes by AMPHORA of the respective genome draft (Additional file 3: Table S4).

To estimate the required sequence depth using a redundancy-based approach, the tool Nonpareil [42] is applied to all samples of interest. Nonpareil provides a projection of the required sequence depth to cover 95% of the sampled biological diversity. This estimation gives valuable insight for sufficient coverage per sample and the proportion of the original diversity which can be expected to be obtained when analyzing respective samples.

## Results

This study assesses the potential of assembly-based methods for analyzing urban metagenome datasets by investigating the performance of different combinations of assembly and binning software. Furthermore, to increase our understanding of these types of datasets and to be able to make better informed decisions regarding the tool selection, we simulated the taxonomic composition based on real sample features and assessed the performance of the selected assembly and binning software.

After quality trimming and removal of human reads, 10–46% (Ø 31%) or 0.04–4.5 (Ø 1.2) Gbp were kept for the Boston samples, 35–82% (Ø 75%) or 1.9–5.2 (Ø4.2) Gbp for the Sacramento samples, and 63–91% (Ø 80%) or 0.05–1.93 (Ø 0.63) Gbp for the New York samples. The low number of remaining reads in the Boston samples was mainly due to the high content of human DNA (up to 84.64%) within those samples. Additionally, up to 55% of the reads in Boston samples still contained adapter sequences, which were also removed in the quality control process. Additional file 4: Table S3 shows the numbers of raw reads and quality-trimmed reads in each processing step.

The remaining reads were taxonomically profiled with Centrifuge [38], using the NCBI nt database as a reference. The Sacramento dataset contained on average 63% unclassified reads, a value that was uniform for all stations and all surfaces (SD 1.68). Additionally, about one fifth of the data was classified as eukaryotic and only about 15% of the quality filtered reads were classified as bacterial. For the Boston dataset the average value of unclassified reads was lower (Ø 52%), but also more variable between samples (SD 11.87) with the lowest number of unclassified reads found in grip samples (27%). The Boston dataset showed also a lower content of eukaryotic and a higher percentage of bacterial reads (Ø 38%). The New York samples had the highest bacterial content with 74% on average together with the smallest fraction of unclassified reads (Ø 23%).

### Assembly

All quality-controlled samples were subjected to assembly. Assembly of Sacramento samples resulted in a total assembly length of 18 to 88 million bp with an average length of 46 million bp for all assemblers. Overall, Megahit resulted in the longest assemblies followed closely by SPAdes, whereas MetaSPAdes created considerably smaller assemblies in total length. Average contig length as well as N50 (minimum contig length to cover 50% of the genome) values were highest in SPAdes assemblies, indicating a more complete assembly with only minor losses in total assembly length compared to Megahit in the majority of the samples (Additional file 5: Table S1). The percentage of quality-controlled reads mapping back to an assembly ranged from 7.7 to 38.2% (Ø 18.8%).

**Fig. 2:**
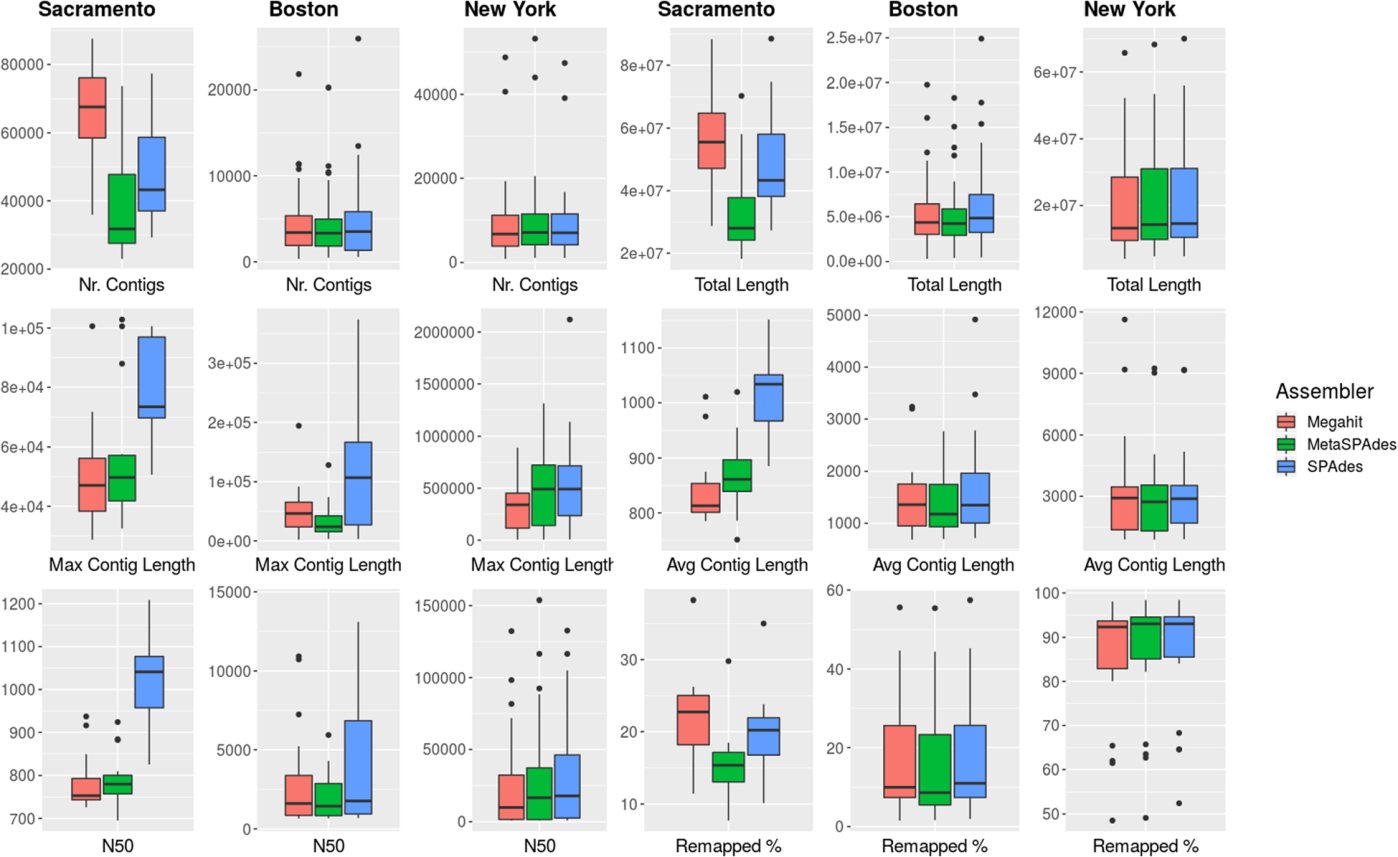
Assembly Statistics for Sacramento, Boston and New York. Assembly statistics for Megahit, MetaSPAdes and SPAdes of all Sacramento, Boston and selected New York samples are shown. Extreme outliers of i.e. a N50 value of over 65,000 for one grip sample from Boston are computed but not shown. Statistics are computed from all contigs above 500 bp in length

Assembly of Boston samples showed substantially more variability in the assembly statistics, as was expected due to the broad range of sequencing depth (0.04–4.2 Gbp after quality control). In contrast to the assemblies from the Sacramento samples, all three assemblers produced assemblies of comparable, albeit short (< 1 mio bp), total assembly length for low-sequencing depth samples. Nevertheless, especially samples from touchscreen surfaces resulted in assemblies with up to 24 million bp of total length. As shown in Fig. 2, SPAdes outperformed the other assemblers for the Boston samples. The ratio of reads mapping back to all assemblies ranged from only 1.56% for samples from seats with very low sequencing depth to up to 57.46% in one grip sample (Ø 17.51%).

New York samples, albeit with similar low-sequencing depth as some Boston samples (0.05–1.93 Gbp), resulted in considerably larger assemblies overall. This is likely due to the much higher bacterial content (Ø 74%) and lower unknown as well as eukaryotic read fraction as determined by classification, resulting in higher sequence coverage of respective bacterial genomes within the community (Additional file 4: Table S3). Total assembly length ranged from 4 million bp to 70 million bp (Ø 22 Mio bp) with SPAdes outperforming the other assemblers again regarding assembly statistics.

A possibility to increase assembly performance of very low abundant species is pooling samples with similar microbial composition to increase coverage of such low abundant species for better assembly results. The disadvantage of pooling samples for assemblies is the potential increase of strain diversity, which in turn severely hampers the performance of assemblies [12]. Due to the substantial higher RAM usage of SPAdes and our limiting computational resources, only pools using Megahit were performed. Samples for Sacramento were pooled according to surface type (A: Bench, B: Ticket machine, C: Platform railing). Pooled assemblies of the three Sacramento surfaces resulted in assemblies of 509, 460 and 473 million bp total length respectively. The total length of pooled assemblies surpassed the sum of all respective single sample assemblies by 169, 130 and 136 million bp for surface types A, B and C, indicating that indeed some additional sequences could be assembled by pooling samples.

### Binning

Assembly of metagenomic sequences results in large numbers of individual contigs that need to be grouped into a genome context. Binning algorithms sort assembled contigs into distinct genome bins by using information like coverage per contig or tetranucleotide frequencies. Ideally these genome bins represent almost complete draft genomes that can represent a pan-genome of a species or a single strain, depending on the quality of the provided assembly. As mentioned in the introduction, high strain diversity is known to decrease the completeness of resulting genome bins [12].

Three different binning methods with promising results in the CAMI Challenge were applied to the respective assemblies of Sacramento, Boston and New York samples, namely CONCOCT [27], MaxBin [25] and MetaBAT [26]. All resulting bins were classified into high, medium and low-quality drafts according to the Minimum Information of a Metagenome-Assembled Genome (MIMAG; Table 1) [32]. The applied metrics are genome completeness and contamination, measured by the presence of single copy marker genes, the presence of rRNA clusters, as well as the presence of tRNAs.

Only medium and high-quality draft bins were considered for further analysis, low-quality draft genome bins were excluded. SPAdes assemblies led to a higher number of high-quality bins compared to the other tested assemblers, with 27, 29 and 30 high-quality bins achieved by MetaBAT, CONCOCT and MaxBin respectively. Additionally, SPAdes assemblies produced 68, 40 and 57 medium-quality bins respectively. The total number of at least medium-quality bins were similar when binning MetaSPAdes and Megahit assemblies, but with a considerably lower fraction of high-quality bins, with only MetaSPAdes/MaxBin achieving 26 high-quality bins, while all other combinations yielded a substantially lower number (Additional file 6: Table S2). CONCOCT and MaxBin tended to bin more rRNA clusters to the same bin with 25/55 and 28/74 high-quality bins from all assemblies holding more than one rRNA cluster for CONCOCT and MaxBin respectively. All high-quality bins resulting from SPAdes/MetaBAT in contrast did not contain multiple copies of 5S, 16S and 23S rRNA clusters, although in some bins more than one 5S rRNA was present (Additional file 2: Table S2).

To determine if multiple rRNA clusters originate from closely related species or represent wrongly binned sequences, we blasted all 16S rRNAs of bins with multiple copies against the 16S ribosomal RNA sequences for Bacteria and Archaea from NCBI. Indeed, additional rRNA copies within a bin originated from distinct taxa. 16S rRNA genes from MetaBAT were assigned to the same taxa as determined by phylotyping by AMPHORA, while the observed multiple 16S rRNA copies by CONCOCT and MaxBin represented wrongly binned sequences (Additional file 3: Table S4).

Binning of pooled Sacramento assemblies provided one more medium (29) and one less high-quality genome draft bin compared to single sample assemblies. Although the number of at least medium-quality genome drafts did not show any considerate increase, the number of bins with high contamination values (> 30% contamination) increased substantially, which was especially true for CONCOCT and less so for MaxBin. As no increase in at least medium-quality bins originating from low coverage genomes was observed, and resulting bins instead showed higher contamination values, we did not further consider this approach.

Only one single sample from Sacramento provided a high-quality draft bin by four different assembler/binner combinations (Sample 4C, platform railing), while all other 18 bins from all assembler/binner combinations of Sacramento samples with sufficient completeness values above 90% and contamination below 5% did lack at least one of the three required rRNAs to be classified as a high-quality draft bin. For these 18 bins, at least 18 tRNAs were predicted. The same could be observed in all genome bins with proper completeness and contamination from Boston and New York samples. The lack of at least one rRNA was predominantly the reason to fail the criteria for high quality genome drafts while a minimum of 18 tRNAs were present.

### Phylotyping

To infer the phylotypes of all high and medium quality bins, AMPHORA2 [29] was applied to every bin. AMPHORA2 uses a phylogenetic marker database of 104 archaeal and 31 bacterial marker sequences to infer phylogeny of metagenomic bins. AMPHORA2 reports a confidence level for each taxonomic level and marker sequence with a successful alignment and therefore assignment. Only assignments with confidence levels above 0.8 (from 0 to 1) were considered and for every bin the lowest taxonomic level with all marker sequences sharing the same assignment is considered. If a bin showed both the presence of archaeal and bacterial sequences, the bin is assigned to the level ‘None’.

**Fig. 3:**
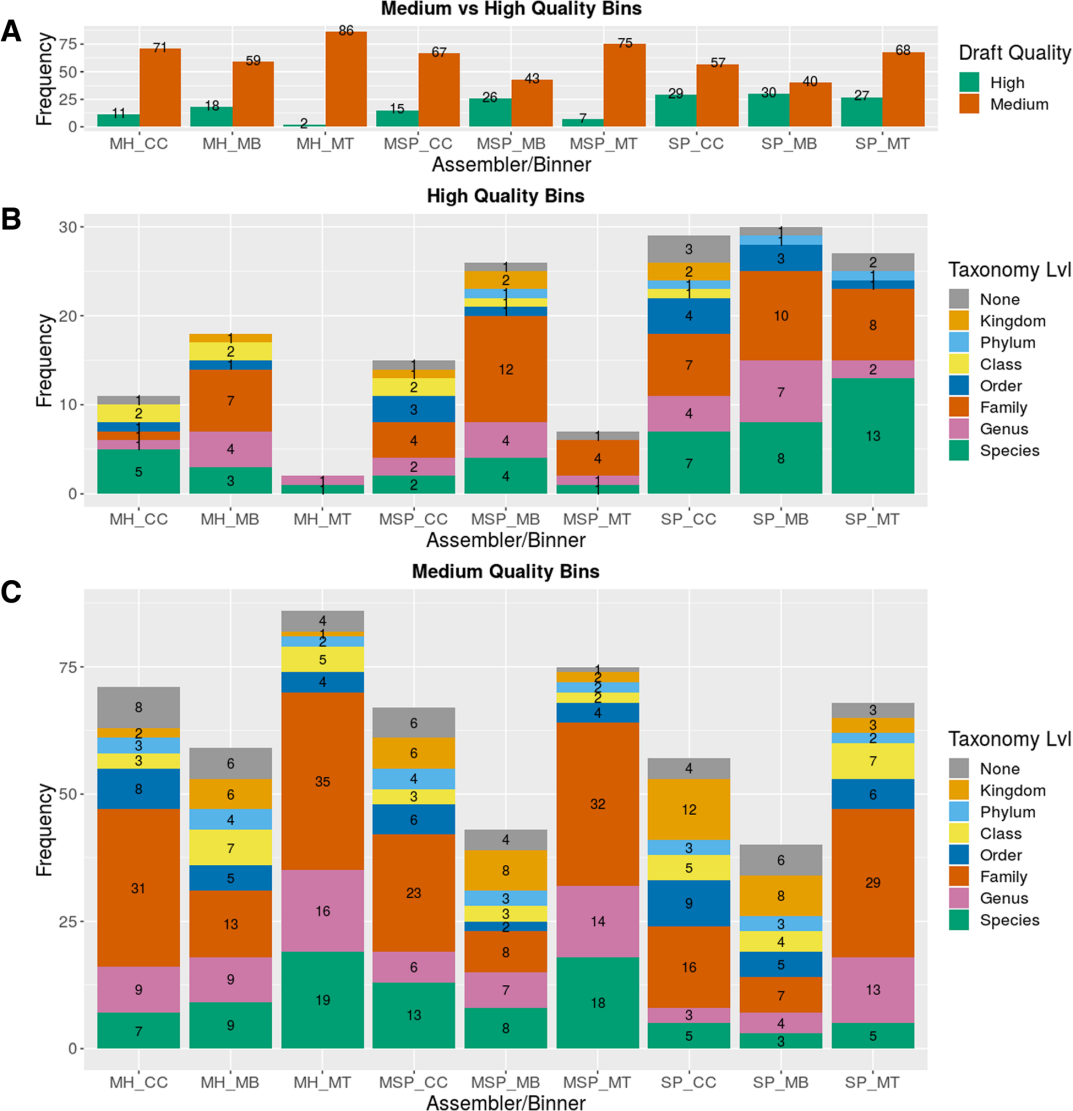
Medium and high-quality bins from assembler/binner combinations. **a** Comparison of medium and high-quality genome drafts obtained from various assembler/binner combinations. **b** Lowest consensus of taxonomic level for all high-quality genome drafts obtained by AMPHORA2. **c** Lowest consensus of taxonomic level for all medium-quality genome drafts obtained by AMPHORA2. MH = Megahit, SP=SPAdes, MSP = MetaSPAdes, CC=CONCOCT, MB = MaxBin, MT = MetaBAT

While all three binning methods achieved similar numbers of high-quality bins from SPAdes assemblies (Fig. 3a), AMPHORA2 reported more bins from MetaBAT with a consensus of all marker genes down to species level than MaxBin and CONCOCT, with 13 bins achieving consensus at species level for MetaBAT compared to 8 and 7 bins for MaxBin and CONCONCT respectively.

This agrees with the results from the 16S rRNA gene analysis, where CONCOCT had the highest number of wrongly assigned 16S rRNA sequences, followed by MaxBin while no multiple 16S rRNAs copies of other taxa from MetaBAT in high-quality genome bins were detected. This indicates less contamination and as such a better consensus of all marker genes at lower taxonomic levels when binning with MetaBAT (Fig. 3b).

While the combination of SPAdes/MetaBAT provided three high-quality bins less than the highest number of 30 bins from SPAdes/MaxBin, it achieved the highest number of at least medium-quality with 95 bins. SPAdes/CONCOCT and SPAdes/MaxBin resulted in 86 and 70 bins of at least medium-quality. The lowest consensus taxonomic level achieved by phylotyping medium-quality bins matched the results of high-quality bins with 47/68 medium-quality bins from MetaBAT achieving consensus at family level or lower, while this was only true for 14/40 and 24/57 for MaxBin and CONCOCT respectively (Fig. 3c).

### Genome analysis of selected bins

We investigated the genome characteristics of bins resulting from the best-performing combination of assembly and binning method (SPAdes/MetaBAT). For the prediction of potential microbial traits from medium to high-quality genome drafts, we applied the PICA framework [36]. PICA cannot only predict traits for complete genomes but allows for most traits also incomplete and contaminated genome sequences as input. We predicted phenotypic traits using the PICA approach and PhenDB [36]. Predicted traits for the *Clostridium difficile* bin from a New York sample show expected traits from Clostridia such as being anaerobe, gram-positive as well as the possibility to form endospores [43] despite low ANI values to the closest strain found by blast. All *Propionibacterium acne* typed bins present expected traits from *P. acne* strains. *P. acnes* is an aerotolerant anaerobic gram-positive bacterium reported in the human skin microbiome [44] as predicted together with phenotypic traits such as recycling of organic phosphorus and degradation of urea (Table 2).

**Table 2:** 27 high-quality genome drafts from SPAdes/MetaBAT

Sample/Bin	Compl.	Cont.	Closest Species	Phenotypic traits	Predicted Proteins	iRep	AAI	ANI
Grip5941 #1	100	0.11	*Propionibacterium acnes*	1, 2, 13, 15, 20, 23	2341	1.41	97.28	99.89
Grip6354 #1	100	0.55	*Propionibacterium acnes*	1, 2, 13, 15, 20, 23	2400	1.45	97.30	99.83
Grip6358 #1	98.9	1.23	*Propionibacterium acnes*	1, 2, 13, 15, 20, 23	2354	1.38	97.34	99.91
Grip6361 #3	98.93	2.34	*Propionibacterium acnes*	1, 2, 13, 15, 20, 23	2507	n/d	96.05	99.97
Pole5898 #1	99.34	0.55	*Propionibacterium acnes*	1, 2, 13, 15, 20, 23	2357	1.42	96.67	99.75
Pole6380 #1	99.12	4.23	*Propionibacterium acnes*	1, 2, 13, 15, 20, 23	2595	n/d	97.07	99.55
Sb5919 #4	99.01	0.66	*Propionibacterium acnes*	1, 2, 13, 15, 20, 23	2349	1.40	97.46	99.81
Sb5948#2	100	0.13	*Propionibacterium acnes*	1, 2, 13, 15, 20, 23	2380	1.48	97.03	99.56
Ts5934#1	99.78	1.75	*Propionibacterium acnes*	1, 2, 13, 15, 20, 23	2441	n/d	96.85	99.76
Ts5963 #1	99.56	0	*Propionibacterium acnes*	1, 2, 13, 20, 23	2366	1.38	97.39	99.89
Ts6363 #1	100	0.11	*Propionibacterium acnes*	1, 2, 13, 15, 20, 23	2390	1.34	96.88	99.75
Ts6367 #1	100	0	*Propionibacterium acnes*	1, 2, 13, 15, 20, 23	2351	1.42	97.12	99.83
Ts6375 #2	99.34	1.86	*Propionibacterium acnes*	1, 2, 13, 20, 23	2382	1.39	96.92	99.93
Ts5059 #2	100	0	*Propionibacterium acnes*	1, 2, 13, 15, 20, 23	2313	1.36	97.47	99.86
Metal8994 #2	99.36	0.14	*Pseudomonas stutzeri*	1, 2, 6, 8–12, 14–18, 21, 23	4151	n/d	95.88	98.03
Metal8994 #5	99.07	0.63	*Enterobacter cloacae*	1–3, 6, 7, 9–12, 15, 16, 19, 20, 23, 24	4405	n/d	93.51	92.02
Metal9078 #4	98.77	2.9	*Pseudomonas stutzeri*	1, 2, 4, 6, 8–12, 14–16, 23	4160	n/d	84.17	84.88
Metal9087 #2	100	0.65	*Pseudomonas xanthomarina*	1, 6, 8–12, 14–16, 23	4048	n/d	86.97	84.58
Metal9150 #4	99.3	1.4	*Clostridium difficile*	2, 9, 11, 13–16, 22, 23,	2818	n/d	78.26	77.02
Metal9150 #5	99.57	0.42	*Stenotrophomonas rhizophila*	1, 4, 5, 7, 9, 10, 12, 14, 15, 19, 20, 23	3937	n/d	86.37	84.58
Metal9150 #7	100	0.26	*Leoconostoc mesenteroides*	1–3, 13, 23	1785	n/d	97.24	99.65
Metal9957 #3	94.52	0.27	*Pseudomonas stutzeri*	n/d	3954	1.51	96.68	98.09
Metal0032 #2	99.83	0.16	*Stenotrophomonas maltophilia*	1, 5–10, 14, 15, 19, 20, 23	4069	n/d	76.15	90.47
MePl9373 #2	99.59	0.2	*Pseudomonas stutzeri*	1, 2, 4, 6, 8–12, 14–16, 18, 23	4157	n/d	84.25	85.30
MePl9832 #7	98.59	3.76	*Gottschalkia acidurici*	2, 9, 12–16, 23	4227	n/d	74.76	85.30
Wood9044 #4	99.47	0.33	*Enterobacter hormaechei*	1–3, 6, 9–12, 15, 16, 19, 20, 24	4352	n/d	97.63	99.04
Wood9200 #3	100	0	*Weeksella virosa*	1, 9, 14, 19, 20, 23	3323	n/d	72.79	74.08

All high-quality genome draft bins from SPAdes/MetaBAT are listed. Sample names are abbreviations of surface names (Sb = Seat backs, Ts = Touchscreen, MePl = Metal/Plastic) and the last four digits of respective SRR ID’s (Additional file 3: Table S2). n/d = values could not be determined as one or more filters failed for calculation. Present number for phenotypic trains indicates a trait being predicted as present (1: aerobe; 2: anaerobe; 3: facultative anaerobe; 4: Type III secretion system; 5: Type IV secretion system; 6: Type VI secretion system; 7: alkane degradation; 8: benzoate degradation via hydroxylation; 9: butyrate producing; 10: chitine degradation; 11: CO assimilation; 12: trimethylamine production via choline; 13: stains gram-positive; 14: bile acid degradation; 15: H2 gas production; 16: self-propelled motion; 17: N2 fixation; 18: fatty acid degradation; 19: hydrolyzing phosphonate; 20: recycles organic phosphorus; 21: oxidizes thiosulfate; 22: produces endospores for persistence; 23: urea degradation; 24: reduces various alpha,beta-unsaturated and nitro compounds)

Additionally, for high-quality genome drafts it is possible to calculate in silico replication rates of near-complete genome drafts applying iRep [37]. The replication rate should not be confused with a dead or alive measurement, as it only measures replication rates based on the difference of coverage from the origin towards the terminus of replication in bacteria. Nevertheless, replication rates might give valuable information about single community members, indicating active replication, while DNA originating from dead bacteria likely fails to provide iRep values due to non-uniform coverage of the genome as expected from living organism.

For all the grip, pole, touchscreen and seat samples from Boston, the majority of bins was assigned to species *Propionibacterium acnes* with ANI values between 99.55 and 99.97. Table 2 shows the number of predicted proteins and their average identity to *P. acnes* of each of the 27 high-quality bins of the SPAdes/MetaBAT combination.

Apart from *P. acnes*, the seatback samples contained medium-quality bins (Additional file 6: Table S2) with the closest homolog being *Micrococcus luteus*, *Streptococcus sanguinis*, and a member of the *Neisseria* genus, identified as *Neisseria sicca* with an ANI value of 96.36. One high-quality genome bin (SPAdes/CONCOCT) from the grip sample SRR3546361 was assigned to the genus Corynebacterium, but a BLAST search on all predicted proteins revealed *Lawsonella clevelandensis* as closest homolog with 78% AAI. The assembled genome might therefore represent a species from the order of Corynebacteriales that is not present in our public databases yet.

For the New York samples high-quality draft genome bins contained *Pseudomonas stutzeri* (ANI 98.03 and 98.09*)*, a denitrifying soil bacterium; *Leuconostoc mesenteroides* (ANI 99.65), a species associated with fermentative conditions; and *Enterobacter hormaechei* (99.04), a human gut bacterium. For other high-quality bins in New York samples, the assigned classification (Table 2) represents the closest homolog found in the NCBI nr database, but not the species found in the sample (indicated by the low ANI values).

### Targeted in silico gold standards

To represent each surface type in Boston, Sacramento and selected New York samples, we picked one sample of each surface type and city for the creation of in silico gold standards with the exception of samples taken from seats in Boston, as sequencing depth of original seat samples was already very low for sufficient assembly resulting in total assembly lengths of at most 5 million bp.

The selected samples were 1A (bench), 1B (ticket machine) and 6C (platform railing) for Sacramento, SRR3546361 (grip), SRR3545898 (pole), SRR3545919 (seat back) and SRR5456367 (touchscreen) for Boston as well as SRR1749044 (wood), SRR1749150 (metal) and SRR1749832 (metal/plastic) for New York.

Between 80.3 and 98.9% (Ø 93.4%) of all classified bacterial reads could be assigned to a reference genome. The number of selected reference genomes ranged from 3067 to 3995 (Ø 3667). Of the remaining few percent, either no reference genome could be assigned (Ø 2.8%) or bacterial reads were only classified to a higher taxonomic level than species level (Ø 3.8%). Resulting gold standards had in average a slight increase of 1% in total number of base pairs compared to the quality-controlled real-life samples despite the loss of about 6.6% non-assigned bacterial reads. This is due to the shorter reads remaining after quality control (minimum read length 70 bp) in real life samples which are counted as a full read. All simulated reads are created with the full read length as observed in respective real samples.

Classification of all reads showed distinct profiles between cities. The total number of different genomes selected for each sample was in the same range in Sacramento (3889 to 3995) and Boston (3434 to 3986), while for New York the numbers were lower (3067 to 3488). A major difference in the composition of selected genomes in all gold standards could be observed in the highest genome coverage of single reference genomes. Using the number of reads attributed to a reference genome and the selected read length, the coverage of each reference genome in a gold standard could be obtained. The maximum coverage of classified reads per selected genome, i.e. the most abundant species classified, was considerable lower in Sacramento with a maximum coverage of a single reference genome between 1.5× and 5.9× compared to Boston with values between 12.8× and 100.5× as well as New York with the highest coverage values of classified bacteria between 24.2× and 196.6×. Only very few classified bacterial genomes were covered above 1× in respective gold standards, with at most 3 to 30 genomes in all gold standards while these genomes often represented multiple strains of the same highly abundant species.

An additional approach to estimate the average coverage of sequences in a metagenomic sample and the corresponding required sequencing effort is the redundancy based approach by Nonpareil [42]. For Sacramento samples, the estimated average coverage was between 0.2× and 0.4× except for sample 4C which was well above 0.5× (Additional file 7: Figure S2). Indeed, sample 4C was also the only Sacramento sample of which a high-quality genome draft bin was obtained.

The same estimates for Boston and New York samples show a consistently higher estimated average coverage per sample compared to Sacramento with nearly all samples reaching values above 0.5× as well as multiple samples from New York reaching saturation with more than 0.95×. (Additional file 8: Figure S3, Additional file 9: Figure S4, Additional file 10: Figure S5, Additional file 11: Figure S6).

To reach an average coverage of 0.95, Nonpareil estimates a required sequencing effort of about 100 Gbp per sample for Sacramento, while only about 10 Gbp were estimated to be sufficient in Boston and even less than 1 Gbp per sample for New York. These estimates can be partly attributed to the highly varying amount of eukaryotic sequences in respective samples, where Sacramento had the highest proportion of sequences classified to plants (using the NCBI nt with Centrifuge), Boston had the highest amount of human sequences and New York the least amount of eukaryotic and unclassified sequences and thereby the highest relative amount of bacterial sequences. These differences likely originate from differing sampling procedures as well as locations, as Sacramento sites were exposed to open air in contrast to underground subway stations.

Following the creation of in silico gold standards, all gold standards are assembled and binned using the exact same workflow as their real sample counterparts. Total assembly length in Sacramento gold standards was reduced by 15% on average (− 39% to + 7%). In contrast, Boston gold standards showed an increase of total assembly length by 31% on average (+ 4% to + 65%) while New York gold standards only showed a slight increase total assembly length on average of 2% (− 17% to + 22%) (Additional file 12: Figure S7). Different assembly statistics are to be expected, as reads classified to a reference strain do not necessarily need to represent this exact strain in the real sample, such as that only parts of the actual strain in the real sample share exact sequences with strains of RefSeq genomes thereby leading to a different assembly performance.

Assembled gold standards provided 12 high-quality and 134 medium-quality genome drafts after binning, while binning of the same original samples resulted in 44 high-quality and 180 medium-quality genome drafts. 5 out of 12 high-quality bins originated from the combination of SPAdes/MetaBAT, with another 3 coming from SPAdes/CONCOCT and the remaining high-quality bins from MetaSPAdes/MetaBAT and MetaSPAdes/CONCOCT providing 2 bins each (Additional file 6: Table S2).

Our assembled and binned gold standards enabled us to investigate misassemblies within our retrieved high-quality genome drafts, as reference genomes sampled with high coverages are expected to be retrieved as a genome draft bin as well. Of these 12 high-quality bins, only a high-quality genome draft for *Leuconostoc mesenteroides* was retrieved by MetaBAT and CONCOCT from SPAdes as well as MetaSPAdes assemblies to be able to compare misassembly values across all four combinations. We selected the reference genome with the highest aligned genome fraction to compare the number of misassemblies, the length of all contigs containing misassemblies as well as the total number of unaligned base pairs as reported by MetaQUAST. Both CONCOCT and MetaBAT bins had a matching best aligned reference genome, namely *Leuconostoc mesenteroides* subsp. *mesenteroides* J18 for SPAdes assemblies and *Leuconostoc mesenteroides* subsp. *Dextranicum* for MetaSPAdes assemblies, both being part of the gold standard for the New York sample SRR1749150. The number of misassemblies were lower for bins using SPAdes assemblies with 12 and 20 misassemblies as well as 97,193 and 196,151 unaligned base pairs for MetaBAT and CONCOCT respectively. MetaSPAdes based bins for the exact same reference genome resulted in 17 misassemblies for both and 106,178 and 180,553 unaligned base pairs for MetaBAT and CONCOCT respectively, although this reference genome was only 3rd best according the genome fraction aligned of all references. The reference genome with the highest alignment fraction for MetaSPAdes even had more than 30 misassemblies and above 1 mio unaligned base pairs for both binning methods. Again, the SPAdes based assembly showed lower misassembly numbers from MetaBAT for the best aligning reference of MetaSPAdes based bins, except CONCOCT performed slightly worse in this comparison again (Additional file 3: Table S4). Overall, the combination SPAdes/MetaBAT showed the least amount of misassemblies compared to both reference genomes with an alignment fraction of 65.4% to 68.7% and the lowest number of unaligned base pairs.

## Discussion

Even though estimates of Nonpareil [42] show an average sequence coverage of only 0.4 to 0.6× for Boston, Sacramento and partially New York, indicating that a major part of the microbial community was not sequenced with sufficient coverage, genome drafts with high-quality draft status could still be assembled and binned from all three cities. Although some of the high-quality drafts were identified by Centrifuge as the most abundant species, like in the case of *P. acnes* in Boston, this was not true for all medium and high-quality draft genome bins. Considering the high amount of human sequences in Boston samples, this finding is not surprising, although we would have expected to see different skin associated bacteria.

Samples having a high proportion of classified reads of a single reference genome, still showed a substantial number of unknown reads when mapping the reads back to the resulting genome draft bin. One pole sample (SRR3545898) provided a high-quality genome draft bin with taxonomic inference of *P. acnes* down to species level in both the original sample (ANI 99.75 to *P. acnes* strain *PA_15_2_L1)* as well as in the in silico gold standard (both SPAdes/MetaBAT, bin #1 each). This species was highly abundant in the sample, with 13.9% of all reads in the original sample and 15.33% in the gold standard mapping to the genome draft bin. Investigating the bin originating from the gold standard, all sampled reads of the most abundant *P. acnes* strain classified mapped to the genome draft bin but so did ten times the number of unclassified reads from the real-life sample which were kept in the gold standard as they were unclassified. Contamination of the genome draft bin was estimated to be 4.18% with a strain heterogeneity of 84.62%, likely originating from the difference of the actual strain within the sample to the reference genome and duplicated single copy genes thereof which could not be separated by binning.

Overall samples from Sacramento, Boston and New York displayed various substantial differences. Sequencing depth was in average nearly four times higher in Sacramento samples together with less than 4% human sequences detected in all samples except Sample 5B and 6C containing 38% and 55% human sequences respectively. In contrast, all Boston samples contained at least 18% (Ø 42%) human sequences while New York samples showed less than 1%. Very high proportions of human sequences might originate from the sampling process, which could also explain the very high abundance of *P. acnes* strains, a prominent member of the skin microbiome [44], in these samples. The high amount of human sequences might thereby lead to the dominating presence of skin microbiome associated bacteria, enabling high-quality genome drafts due to their high abundance.

Higher sequencing depth together with less human sequences in Sacramento samples did not immediately lead to higher average coverage of single bacterial species. In fact, estimated average coverage according to Nonpareil estimates was even lower. This could originate from a substantial part of low abundant microbial species being heavily underrepresented as well as a higher diversity of sequences with eukaryotic origin such as plants which would require substantially more sequencing depth to be covered multiple times compared to bacteria. As New York samples had the highest relative proportion of bacterial sequences, estimated required sequence depth was one to two magnitudes lower as in Boston and Sacramento, also represented by the higher number of obtained high-quality genome bins from these samples.

In all samples, we observed the presence of required 5S, 16S and 23S rRNAs to be a major eliminating factor for bins to achieve the level of high-quality genome drafts. We believe this to be caused by the presence of multiple closely related strains hampering assembly and binning. A large number of different strains from e.g. skin microbes present in urban environmental samples can be expected from a high number of different people introducing different strains to the microbial communities of the sampled surfaces. Nevertheless, all three binning methods behaved differently when binning rRNA regions. None of the high-quality genome draft bins originating from MetaBAT contained more than one copy of all three 5S, 16S and 23S rRNA, while in rare cases one of the three rRNAs was duplicated. In contrast, high-quality genome drafts originating from CONCOCT and less so from MaxBin tended to contain multiple copies for each of the three rRNAs, of which additional 16S rRNA copies predominantly originated from other species when aligning them against the 16S ribosomal RNA sequences (Bacteria and Archaea) from NCBI (Additional file 3: Table S4).

SPAdes produced the largest assemblies as well as the highest number of resulting bins. As already reported by van der Walt et al. [21], MetaSPAdes seems to have more difficulties assembling very low coverage genomes compared to SPAdes and Megahit, while SPAdes is reported to produce more misassemblies When analyzing our resulting bins from urban metagenomes, we did indeed detect misassemblies in genome bins of in silico gold standards based on SPAdes assemblies, though we did also detect an even greater amount of misassemblies and unaligned base pairs in MetaSPAdes based bins (Additional file 3: Table S4), especially when comparing a high-quality draft from the same sample for which all combinations of SPAdes and MetaSPAdes as well as MetaBAT and CONCOCT provided high-quality genome drafts for *Leuconostoc mesenteroides*. Although MetaSPAdes/CONCOCT created the only high-quality genome draft bin for *Stenotrophomonas maltophilia* from the New York sample SRR1749832 without any misassembly, analysis by MetaQUAST also showed 212,908 unaligned base pairs (Additional file 3: Table S4). The overall presence of misassemblies is not surprising, as multiple strains from the same species were ultimately binned into the same genome-draft, as the binning methods were not able to separate strains from the same species with similar abundances. The differences between the strains together with potential assembly errors lead to the reported misassemblies as well as unaligned base pairs likely originate from sequences of other strains of the same species. Multiple strains for highly-abundant species such as *P. acnes* are to be expected in urban metagenomes as they likely originate from multiple humans interacting with respective surfaces.

MetaSPAdes resulted in the highest number of medium-quality bins closely followed by SPAdes based on gold standards while in real samples SPAdes had slightly more medium-quality bins than MetaSPAdes. Megahit provided bin numbers within the same range, although substantially less high-quality bins were retrieved in both real samples and gold standards.

Pooling the samples to increase sequence coverage of rare species within the metagenomic community did not result in an increase of obtained bins of such species compared to single samples. One of the reasons for the lack of improvement, could be that the surface type is not a determining factor for species composition, and that the diversity of the sample is increased by pooling, leading to the detrimental effect of increased diversity overpowering the increase of sequence coverage for certain species for assembly and binning efficacy. This would explain the massive increase of contamination within resulting bins, where binning methods struggle to separate closely related species and merge them into single bins. These high contamination values prohibited many resulting bins to achieve at least medium-quality draft status.

By obtaining high-quality genome drafts from the best performing combination of SPAdes/MetaBAT we could provide genome drafts from various species. While Boston samples resulted in bins originating mainly from *Propionibacterium acnes* strains, New York samples showed a higher diversity of bacterial species of which high-quality genome drafts could be obtained (Table 2). Samples from Sacramento did not result in a large number of high quality bins, but a higher number of different species could be identified in medium-quality bins (Additional file 6: Table S2). Some of these species are also associated with human skin as well as oral or respiratory tract microbiome, while others are more ubiquitous such as the only high-quality bin resulted from a ticket machine sample (4C) identified as a species of the Halomonadaceae family. This family is made up of extremophile organisms being able to withstand high salt concentrations.

Such genome drafts enable detailed analysis of single community members up to comparative genomics, which go beyond the scope of this study. However, we want to showcase additional analysis steps of genome draft bins regarding urban metagenomics.

For environmental samples such as urban metagenomics, it is not only interesting to know who is there and what they do, but also who is still living, and which species might just be transferred to certain surfaces and die off subsequently. Consistently positive iRep values of the same species such as obtained from *P. acnes* strains in Boston samples indicate that these strains still can replicate on respective surfaces and thereby likely at least survive for a short time. Absence of iRep values nevertheless cannot be used to conclude that respective strains were dead as failure of multiple filters for iRep calculation can have various origins such as too low coverage or interference of DNA from dead cells while some bacteria are still alive leading to non-uniform coverage patterns.

Beside replication rates of single community members, phenotypic traits of identified, assembled and binned species can help us to understand the role and activity of certain species within a sample or environment such as the presence of anaerobe strains closest to Clostridia with the potential to form endospores.

During the creation of in silico gold standards, only classified reads are considered. As classification is database dependent, it is likely that a number of unclassified sequences still belong to a close relative of strains within the database and are subsequently not simulated. On the other hand, if a reference strain is assigned a very high number of classified reads, it is also possible that not all reads originate from the exact same strain but another unknown strain of said species is so close to the reference strain, that a high number of reads are classified to said reference. This would reduce the actual strain diversity in the gold standard of said species and therefore increase assembly performance.

Nevertheless, investigating high-quality genome-drafts derived from reference sequences in in silico gold standards together with the unclassified sequence part of the original sample allowed us to determine a large proportion of unclassified reads to originate from known species, but representing unknown strains.

## Conclusions

In this study we show that, even for datasets with low sequencing depth and high diversity, assembly-based methods can provide valuable results that complement read-based or marker gene-based approaches and allow the community to gain additional insight into the dataset as well as critically assess taxonomic profiles for these types of datasets. Assembled genomes allow a much more detailed level of functional analysis, phenotypic trait prediction of single community members and a clear link between specific markers and the species as well as integration of sequences into the analysis which are not represented in databases for read-based methods. In the investigated datasets we were able to create high-quality genome drafts predominantly from *Propionibacterium acnes* for Boston samples, as well as additional taxa such as *Pseudomonas stutzeri, Stenotrophomonas maltophilia* from New York. Sacramento samples, despite providing more sequence depth than most New York samples, provided inferior results, mainly due to substantial higher eukaryotic and lower bacterial sequence fractions, also leading to very high estimates for required sequence depth by Nonpareil. For many species, coverage was not sufficient for proper assembly and binning, nevertheless many medium-quality genome drafts could still be obtained for these taxa. Using an assembly approach allowed us to predict genes for all our genome bins as well as investigate the traits they contain. Additionally, high-quality genome drafts can be used to calculate the replication activity of respective species within the microbial community.

Considering the number of high-quality draft genomes, correct rRNA cluster assignments, consensus of phylogenic marker genes and misassemblies, the combination of SPAdes and MetaBAT provided the best results for the presented urban metagenomic datasets. We could demonstrate the use of sample-specific in silico gold standards to select appropriate methods for assembly and binning of metagenomic data, with matching tool performance in real samples compared to the assessment performed with gold standards.

In case of limiting computational resources, assembly by Megahit is a viable option, due to considerable lower computational resource requirements [13, 21]. Using Megahit assemblies, MaxBin and CONCOCT provided more high-quality bins than MetaBAT, although MetaBAT still provided the highest number of overall bins including medium-quality.

Methods were to some part complementary as well, with one binning method providing bins for a species in high-quality, where another method only achieved medium-quality for the same taxa, again demonstrating the difficult choice of an optimal tool setup for each analysis.

The use of in silico gold standards helps to uncover the properties of specific datasets and could be used to model differences between datasets as well as enabling further investigations into specific biases of methods focused on the sample composition of interest.

However, to fully unlock the potential of assembly-based methods for urban metagenome studies in order to uncover the yet hidden part of the urban metagenome, we clearly need to improve the sequencing depth, so that we may understand the complexity and dynamics of the microbial communities in this environment.

## Reviewers’ comments

### Reviewer’s report 1

Craig Herbold, University of Vienna

The manuscript by Gerner et al. outlines an effort to identify currently available tools that are suitable for reconstructing metagenome-assembled genomes (MAGs) from urban microbiome metagenomes. These datasets are typified by high diversity and low sequence coverage, which complicate assembly and genome binning. To identify suitable tools, the authors used combinations of three assembly tools and three genome binning tools and evaluated which combinations of assembly and binning tools produced the highest number of high- and medium-quality MAGs that could be confidently classified. The authors used two sets of data for evaluation: 1) Urban microbiome metagenomes generated as part of the 2017 CAMDA challenge and 2) innovative in-silico mock metagenomes that closely mimic the urban microbiomes. From their results, the authors identified SPAdes as the best assembly tool and superficially similar performance from Metabat and CONCOCT as the best binning tool. Bins reconstructed using Metabat however outperformed CONCOCT in terms of phylogenetic consistency based on single-copy marker genes and the presence of homogenous rRNA sequences. With their mock communities, the authors show that binned genomes probably contain unique sequence, as compared to genomes present in current databases and the use of these bins can result in additional taxonomic and/or functional assignment of raw sequence data. I found the study to be an interesting addition to the literature on assembly and binning practices, particularly for researchers interested in studying the microbiome of highly diverse, low-biomass environments.

**Reviewer comment:** I found it quite interesting and counter-intuitive that the SPAdes assembler run in single-genome mode outperformed assembly tools specifically designed for metagenomic datasets. The van der Walt, 2017 study cited by the authors observed something similar, however that manuscript specifically noted that SPAdes tends to produce misassemblies when used on complex metagenomic datasets. In the current manuscript, the authors do not address this seeming contradiction. How can an assembler be the best assembler for complex, low coverage datasets if it is also expected to produce misassemblies? What evidence do the authors have for or against co-assembly of closely related strains? Do the genomes reconstructed represent a clonal population? I would urge the authors to directly confront these questions and report to the interested reader why misassemblies may be expected from highly complex datasets and more importantly why the existence of misassemblies might either be ameliorated through binning and/or represents an acceptable trade-off in the current use case. A thorough discussion on this particular aspect of the study would go far in providing useful advice to researchers choosing appropriate tools for their own urban microbiome studies.

Author’s response: *We thank the reviewer for the suggestions. We extended the analysis of high-quality genome drafts from gold standards with known genomes to incorporate misassemblies as well. For our presented data, the combination of SPAdes and MetaBAT provided genome draft bins with the least number of misassemblies and unaligned base pairs, strengthening our former conclusion. We address misassemblies in the discussion as well, explaining why they are expected in the current use case.*

*Applying co-assembly of multiple samples from the same surface origin did not improve the quality of retrieved bins but resulted in considerably higher contamination within resulting bins. We strengthened this statement in the manuscript and did not continue this approach due to this result. We believe the separate samples to be too different to benefit from co-assembly.*

**Reviewer comment:** The innovative use of the mock-communities by the authors is extremely interesting and warranted, however the presentation comes across as overly complex. I urge the authors to revise all sections that deal with this aspect to be clear and succinct. It is innovative in its acknowledgment that the background of non-bacterial sequence can complicate the assembly of Bacteria and that the inclusion of this background places simulated bacterial reads in a natural setting. Furthermore, the mock-community analysis illustrates that de novo assembled bins of Propionibacterium acnes, for example, provide specific genomic information beyond what exists in the collection of Propionibacterium acnes genomes available in pre-existing (mapping) databases. This highlights a key disadvantage of non-assembly based metagenomic analysis that is overcome through the inclusion of sample-specific MAGs. If presented more clearly, these findings would be more efficiently communicated.

Authors response: *We have revised the respective sections and updated the Supplementary Figure 1 for a better representation of the applied workflow to explain more clearly our approach and the key advantages of assembly-based methods.*

**Reviewer comment:** Lines 562-603: discuss general biological aspects of taxa for which MAGs are generated but I am not sure why it is relevant. The information provided does not seem to be specific to the MAGs in this study and could have been deduced without any assembly or binning. Cases in which the authors have identified an unexpected function assigned to MAGs belonging to a particular taxonomic lineage should be clearly described, but there is no reason to list features identified in the MAG that are identical to functions in reference organisms. Furthermore, it would be quite interesting if the authors explored the portion of the assembled genome that had been assigned to P. acnes MAGs, for instance, but which are not represented by existing database entries, the existence of which can be inferred by the mapping to the MAGs by ~1.4% of reads that were not classified as bacterial reads (line 511). These genomic regions are the new data that the authors assigned to P. acnes, and it is this portion of the genome that should be explored explicitly to infer novel functions for this taxon. This sort of analysis would identify a clear and tangible advantage of assembly/binning over standard mapping approaches.

Authors response: *We shortened sections discussing general biological aspects as they are indeed not the main focus of this study. Nevertheless, we believe that the provided, albeit limited, biological aspects of our resulting bins to be helpful to place our results into the context of urban metagenomes. A detailed analysis of novel functions for separate bins and unknown genome regions of resulting bins would indeed be very interesting, although goes beyond the scope of this study, aiming to assess current assembly and binning methods for urban metagenomes while giving an outlook into further possible analysis.*

**Reviewer comment:** Generally, I would disagree with the use of AMPHORA classification as sufficient for assignment of a MAG to a particular species. Given the AAI values reported in Supplementary Table 2, it is likely that the species have been accurately identified, particularly for P. acnes. AAI values have not been thoroughly evaluated for species demarcation however and the authors should report ANI values as well. I would strongly urge the authors to follow the recommendations by Konstantinidis et al., 2017 (doi:10.1038/ismej.2017.113) for the taxonomic evaluation of MAGs to known species and genera.

Authors response: *We thank the reviewer for the suggestion. We added respective ANI values for the closest relative identified by BLAST as recommended by Konstantinidis et al. For completeness and contamination criteria, we chose to apply the MIMAG standards instead being part of the Minimum Information Standards framework.*

**Reviewer comment:** Based on the science, my impression of the manuscript was positive, however the presentation of the manuscript was generally unfocused. The manuscript should go through at least one round of serious revision and each section should be streamlined to focus only on the main messages of this study. The introduction does not highlight the aspects of the CAMI challenge which directly influenced the choice of assemblers and binners tested in the current manuscript and does not introduce and explore the way that read-mapping approaches differ from assembly/binning approaches. These differences however are key to the argument that assembly and binning can identify novel features of genomes that would be lost through mapping techniques.

Authors response: *We extended the introduction regarding the CAMI Challenge and advantages of assembly over read-bases methods. The whole manuscript was streamlined to convey the key messages more clearly.*

**Reviewer comment:** It would be helpful to report preprocessing statistics in a separate supplementary table, apart from Supplementary Table 1. As is, Supplementary Table 1 is very confusing.

Authors response: *We thank the reviewer for the suggestions, preprocessing statistics are now split out of Supplementary Table 1 and moved to Supplementary Table 3.*

**Reviewer comment:** Lines 27-30 are confusing. The comparison of 14 high quality bins on one hand with 36 medium quality bins doesn't tell me much It would be more clear to report the number of high-quality and medium-quality bins under each combination. 14/18 High/Medium-quality bins for SPAdes and MetaBAT compared to 13/27 High/Medium-quality bins for SPAdes and Concoct.

Authors response: *The result section was revised to communicate key results more clearly. The ratio of High/medium quality bins for respective combinations can be seen in Figure 3A. Reported numbers changed, as we were able to include additional data.*

**Reviewer comment:** Lines 30-32 indicates that novel species were binned but the necessary ANI calculations were not conducted to make this claim.

Authors response: *Required ANI calculations have been added to the respective sections.*

**Reviewer comment:** Line 31: What does “good” refer to here? Be more specific.

Authors response: *We changed the wording to medium and high-quality bins.*

**Reviewer comment:** Lines 35-36 consider rephrasing “parts of unclassified reads”. Is correlate the right word here?

Authors response: *We removed the sentence in questions from the abstract and described all mapped unclassified reads more detailed within the manuscript.*

**Reviewer comment:** Lines 39-42 Make the conclusions clearer.

Authors response: *The conclusions were rewritten for better clarity.*

**Reviewer comment:** Lines 51-59: This could be summarized into one to two sentences and still communicate the relevant background.

Authors response: *Respective parts were shortened in the introduction.*

**Reviewer comment:** Lines 64-79: include additional references to support statements made.

Authors response: *We added references from the MetaSUB Consortium to respective statements and rewrote the section.*

**Reviewer comment:** Lines 123-128: Please specify additional information: 1) what minimum contig length was allowed for inclusion into metagenomic assemblies? 2) which minimum contig length was allowed into each binning tool? 3) was binning performed using tetranucleotide frequencies and abundance, and if abundance, how many and which reads sets were mapped to assemblies to produce abundance profiles?

Authors response: *All additional information was added to respective method sections.*

**Reviewer comment:** Lines 168-190: Direct readers to the supplementary table that summarizes numbers of raw reads and number of reads retained after each preprocessing step. Consider separating this information out of the current Supplementary Table 1 and create a new table just to summarize filtering/mapping statistics.

Authors response: *Filtering and mapping statistics have been moved to separate tables with according references in the manuscript.*

**Reviewer comment:** Lines 194-201: This should be part of the introduction, not part of the Results.

Authors response: *The part was moved to the introduction.*

**Reviewer comment:** Lines 228-230: Are these sums higher or lower than the sums of relevant individual assemblies? Did you get more data using the combined assembly? This is intriguing and could be discussed more.

Authors response: *We added more detailed comparisons including the sums of all single individual assemblies compared to respective pools. In the result section for Binning we report no increase in at least medium-quality bins although contamination of resulting bins increased substantially. Due to this observation, we did not pursue pooled samples further.*

**Reviewer comment:** Lines 272-274: Are the multiple rRNA operons assigned to the same bin with CONCOCT identical or near identical to one another? In other words, does this result indicate good or poor performance?

Authors response: *We aligned multiple 16S rRNAs from CONCOCT bins showing that they stem from different species and thereby represent poor performance. Results have been added to Supplementary Table 4 and in the manuscript.*

**Reviewer comment:** Lines 335-346: I do not see what iRep calculations add to the manuscript.

Authors response: *We believe replication rates to be an interesting aspect of urban metagenomes, as it is unknown which species might still replicate on respective urban surfaces. We changed our wording to convey this more clearly.*

**Reviewer comment:** Lines 348-349: Is this 16S strain-based analysis reported somewhere in the manuscript that I missed? I would agree that the bins are P. acnes, but only because the predicted proteins are >96% identical to the reference P. acnes, which is a much stronger argument than the classification by AMPHORA. Without showing the results of the strain-level analysis, I would probably leave this statement out.

Authors response: *The respective section has been shortened, removing the statements while adding 16S rRNA analysis to respective gold standards which are now added to the manuscript.*

**Reviewer comment:** Line 354: These are percentages, not the numbers of predicted proteins.

Authors response: *We changed the word from numbers to percentages.*

**Reviewer comment:** Line 361: Lawsonella is a genus in its own right. Not a member of the genus Corynebacterium.

Authors response: *We thank the reviewer for pointing this out, the statements have been changed accordingly.*

**Reviewer comment:** Line 363: It is highly unlikely that the bins are Variovorax paradoxus with only 69% amino acid identity.

Authors response: *The statement has been removed while shortening said section.*

**Reviewer comment:** Line 368: This is a marginal case in terms of claiming that this bin is from a species of Moraxella or is specifically Moraxella osloensis.

Authors response: *The statement has been removed while shortening said section.*

**Reviewer comment:** Lines 389-487 - parts of this section should be moved to introduction or methods. It is a disproportionate amount of space to spend on this aspect of the study.

Authors response: *Respective sections have been moved and shortened to introduction and methods.*

### Reviewer’s report 2

Serghei Mangul, University of California, Los Angeles

**Reviewer comment:** Definition of in-silico mock community is misleading. Mock community has a very specific definition. Due to the complexity of the biological system, it is impossible to obtain the ground truth in many applications. In these cases, instead of obtaining the golden standard, one can design a mock community (often referred as a synthetic mock community) by combining in vitro titrated proportions of community elements. The most popular mock communities are prepared as mixtures of known microbial organisms. What is presented in this paper, is simulated gold standard. Please refer to MANGUL, SERGHEI, et al. “Towards Reproducible, Transparent, and Systematic Benchmarking of Omics Computational Tools.” Open Science Framework, 12 June 2018. Web. https://osf.io/p8yd9 for definitions and types of gold standards.

Authors response: *We thank the reviewer for pointing this out. We changed all occurrences of mock communities to in silico gold standards and cited the respective publication for the definition.*

**Reviewer comment:** Line 94. Experimental mock community data needs to be distinguished from simulated microbial community (referred as mock community on line 94)

Authors response: *According sections were rewritten, and the naming of gold standards clarified accordingly*.

**Reviewer comment:** The already nice introduction can be strengthened by mentioning the effect of blood microbiome on the mental disorders: Loohuis, Loes M. Olde, et al. “Transcriptome analysis in whole blood reveals increased microbial diversity in schizophrenia.” Translational psychiatry 8.1 (2018): 96.

Authors response: *We thank the reviewer for the suggestion and added the reference to the introduction.*

**Reviewer comment:** Authors do a nice work investigating of de novo assembly to reveal the community composition. NY samples were excluded due to a low coverage. As a principle of concept, it would be nice to show that indeed low coverage samples are not suitable for genome assembly. Author may consider selecting several samples and run the proposed pipeline.

Authors response: *We were able to analyse randomly selected samples from New York and retrieve high-quality draft genomes. We added all results to the manuscript and discussed our findings in respect to sample composition and coverage for successful assembly and binning.*

**Reviewer comment:** Paper mentioned the recent benchmarking paper published in Nature Methods by Sczyrba et al.., which is purely based on simulated data. It should be noted in the manuscript that simulated data is not able to capture true experimental variability and will always be less complex than real data. It is preferable such data to be used as a complementary to the real experimental gold standard. Please refer to : MANGUL, SERGHEI, et al. “Towards Reproducible, Transparent, and Systematic Benchmarking of Omics Computational Tools.” Open Science Framework, 12 June 2018. Web. https://osf.io/p8yd9

Authors response: *We thank the reviewer for the suggestion and included the publication into the introduction and rewrote according sections to clarify our approach. We added explicit notes that our simulated data as other benchmarking approaches are not a true representation of experimental and the complexity of real data but an approximation.*

**Reviewer comment:** Line 104. Some Boston samples have fewer reads that NY one. Were those samples excluded?

Authors response: *No Boston samples were excluded, although very low coverage samples from Boston did not provide sufficient assemblies for subsequent binning. Values are reported in Supplementary tables and we clarified according result sections. New York samples were added to the analysis for comparison.*

**Reviewer comment:** Line 143. Targeted mock community. The word targeted is misleading in this sentence. Mock community is targeted by design. This needs to be rephrased or explained.

Authors response: *We rephrased and extended explanation of according sections.*

**Reviewer comment:** Line 186. Paper report portion of reads classified as eukaryotes. Were those only cell cell eukaryotes? Analysis of single cell needs to be distinguish from plants.

Authors response: *Eukaryotic sequences were determined by classification of all sequences from a sample against the NCBI nt with Centrifuge. Sacramento showed a higher proportion of sequences originating from several plants compared to Boston with a higher proportion of human sequences, according statements have been modified in the result section. We did not investigate the eukaryotic fraction further, as sequencing depth was far too low for extensive eukaryotic analysis using assembly-based methods and thereby going beyond the scope of this study, instead we focused on the prokaryotic fraction of urban metagenomes.*

**Reviewer comment:** In the introduction authors mention host-microbiome interactions, how this is different from host-pathogens interaction (a more common term). Context needs to be provided

Authors response: *We added context to the corresponding section, meaning many human-microbiome associated bacteria found in urban metagenomes represent commensal bacteria and not necessarily pathogens.*

**Reviewer comment:** Line 131. Citation is needed to support criteria for high quality genomes.

Authors response: *We added the required citation to the respective sentence.*

### Reviewer’s report 3

Yana Bromberg, Rutgers University

The manuscript addresses an important problem of properly selecting tools for the analysis of urban metagenomes. The authors had done a significant amount of work in trying to assemble, pool, functionally and taxonomically annotate, and otherwise evaluate the metagenome data from the CAMDA 2017 (Boston and Sacramento) experiment. Their report is relevant for anyone attempting similar exercises on somehow similar sequencing data. A key finding from the study is that different combinations of tools greatly alter the possible outcomes. Curiously, though, the authors also find that functional/phenotypic annotations of even the different bacterial species identified, are similar. To this reviewer, this finding suggests that assembly may not be strictly necessary in metagenome analysis... particularly if the purpose of the analysis is to figure out the functional abilities/biomarkers of the microbiome. To this end, tools such as MG-RAST and mi-faser could be used with significantly less effort. These tools could also do a good job on low coverage samples (like NYC that was excluded in this case). Of note is also the authors' finding that the mock communities that they had created were less well assembled than the original communities. The authors state in their discussion that this is likely due to the limited nature of bacterial reference genome databases. This is a very solid and sound finding, which I would like to support with further suggestion that it is hard to study the currently uncultrable bacteria (read microbiome community members) using what we know about the inherently different (although overlapping) set of currently culturable bacteria.

**Reviewer comment:** It is unclear how the extensive collection of microbiome analysis tools had been selected for this study. It would be great if the authors could summarize the complete state of this field and reason for their selections.

Authors response: *We thank the reviewer for pointing this out and extended our reasoning for tool selection, which is primarily based on the extensive CAMI Challenge as well as additional Publications in the field. All citations have been added to clarify our reasoning for tool selection. A summary of the complete state of the field goes beyond the scope of this study.*

**Reviewer comment:** Please clarify if your findings can be used to argue that the Minimum Information criteria of the high-quality draft assembly can be loosened in terms of rRNA presence, when other terms are satisfied.

Authors response: *We believe the requirement for rRNA presence provides a proper criterion for genome-drafts above 90% completeness and below 5% contamination. We did detect a tendency of falsely binned rRNA sequences to occur, especially with too many rRNA clusters being combined into one genome bin while still conforming the high-quality criteria, the lack of respective rRNAs is thereby an effective filter to retrieve bins of sufficient completeness. Without respective rRNAs, widely applied 16S rRNA analysis with a massive amount of data available for comparative analysis could not be performed for respective genome drafts, being reason enough in our opinion to classify such bins as medium-quality.*

**Reviewer comment:** I am very curious if high quality bins can be extracted from one combination of tools (as described in Figure 2, for example), while medium quality bins from another? What effect on our ability to identify species level organisms would that have?

Authors response: *The total numbers of medium and high-quality bins were roughly similar ranging between 69 and 95 where MetaSPAdes provided the lowest numbers together with respective binners (see Supplementary Table 2). In general, we could observe an increase of medium-quality bins if a very low number of high-quality bins were achieved (as was the case for Megahit based assemblies), indicating overall less bin quality in terms of the MIMAG criteria.*

*For species identification of respective bins, a low contamination value is more important than completeness, as contamination leads to ambiguous assignments which are hard to resolve, hampering species level assignments. Thereby, pure, but incomplete medium-quality draft genomes might still be phylotyped to species level while complete but contaminated bins will result in a higher level of taxonomic consensus assignments.*

**Reviewer comment:** Also, how much overlap between the species that were identified were there between different tool combinations?

Authors response: *Species for which we could retrieve high-quality genome drafts were detected by other tool combinations as well (e.g. P. acnes strains were found by all combinations), although with varying bin quality, occasionally resulting in only medium-quality bins due to more extensive contamination or less completeness such as was the case for bins phylotyped to Enterobacteriaceae from the gold standard based on the New York Sample SRR1749044, for which only SPAdes/CONCOCT achieved a high-quality genome draft, while the other combinations failed high-quality level due to lacking rRNA sequences. We did not perform extensive analysis to identify the correct species for all medium-quality bins but focused on high-quality draft genomes. Only for about 15% of resulting bins, species level assignments agreed (i.e. were present in all combinations of one sample) over all tool combinations. In many cases although, taxonomic inference (by AMPHORA) did not reach species level but genus, family or higher levels of taxonomy although the same species likely was present.*

**Reviewer comment:** Page 4: “no other study tried to accomplish assemblies of urban microbiomes so far” - still true?

Authors response: *This is still true to our knowledge, there were several studies about urban microbiomes (even as recent as July 31*^*st*^*, 2018 by Kang et al. [8]) as provided in the CAMDA challenge which applied read-centric methods and are partially cited in the introduction. We are not aware of an assembly and binning based study of urban metagenomes from the CAMDA challenge or similar urban metagenomic data to date.*

**Reviewer comment:** Page 4: Definitions/clarifications for “purity of the resulting bins”, “microbial dark matter”.

Authors response: *Respective terms were either removed or replaced to clarify the statements.*

**Reviewer comment:** Page 5: Clarify: read length for “Boston was 101bp” - do you mean the average length?

Authors response: *Changed wording to original read length, read length of real data samples from Boston was meant as all reads had a length of 101bp before quality control was applied.*

**Reviewer comment:** It is unclear to me why figure 2 combines Sacramento and Boston data. Was there no city specific signal to talk about?

Authors response: *Former Figure 2, now Figure 3 shows the general behaviour of assembler/binner combinations focusing on method performance for tool selection. Specific city patterns are discussed in the condensed section about biological features, like the dominating abundance of P. acnes strains in Boston samples and a higher diversity in New York.*

**Reviewer comment:** In creating mock communities, could one benefit from taking random organisms from higher level taxa to represent those for a higher taxonomic coverage?

Authors response: *We thank the reviewer for the suggestion, although we believe random sampling of higher taxa would contrast our aim to mimic the original sample distribution as close as possible, deducing reference genomes from the sample composition resulting from classification of all sequences. Introduction of random organism would additionally pose the problem to decide on respective abundance distributions, number of closely related strains and so forth as these ratios do have a major impact on binning and assembly performance as reported in the CAMI challenge due to macro and micro diversity of bacterial species and strains.*

**Reviewer comment:** The Nonpareil estimates of 100Gbp per sample for "good" coverage of higher diversity microbiomes seems unreasonably large given the authors' own experience (page 9) with assembly compute resource limitations. What would be the proposed plan of action in this scenario? Here, I'd like the authors to once again consider that analyzing reads can arguably give more detail (in a shorter and less time/compute intensive frame) regarding microbiome function, if not provide the members “directory.” This is somehow different from one of the paper's conclusions on benefits of assembly and I believe the paper could be more complete with comments on this topic.

Authors response: *We extended our conclusions based on Nonpareil estimates, as a high amount of eukaryotic fraction immediately requires substantial more sequence depth for decent coverage compared to the added New York samples with a higher bacterial read fraction, resulting in required sequence depth estimates about two magnitudes lower.*

*A key difference between analysis of separate reads and assembly-based method is the acquired information about single community members and their separate functions based on the genomic information of a single member of the community as well as the retrieval of (near) complete genomes for additional comparative analysis. Although read-based centric methods are significantly cheaper in terms of resource cost for analysis, ultimately, they provide different levels of results.*

**Reviewer comment:** Please proofread the document. While it reads ok, it would benefit from small changes like the ones I highlight below (there are many more, but I don’t want to continue focusing on language).

Page 7: “methods for urban metagenome datasets” --> methods for ANALYZING urban

metagenome datasets; age 8: Please check your plurals “A wide range of assembler” → “Many assemblers” “that assemblerS” “Three different assemblerS”; “demonstrating a better performance” → better than what?; “were selected for the shotgun metegnome datasets” → “were selected for the assembly of the shotgun metegnome datasets”.

Authors response: *We thank the reviewer for the corrections and have implemented them as far as according sections have not been rewritten.*

## Additional files

Additional file 1: Textfile with commands of tools applied. (TXT 5 kb)

Additional file 2: **Figure S1.** Workflow for in silico gold standards. To create targeted in silico gold standards, all reads from a metagenome sample are classified. All reads classified to a taxonomic level or lower, i.e. bacterial, are simulated in silico in the exact same read counts from respective reference genomes. All simulated reads are then used to replace all classified bacterial reads, creating a gold standard maintaining composition of read errors, contamination and other characteristics of a specific sample, while providing known truth about a taxonomic domain of interest. All real samples together with their gold standard counterparts are processed in parallel using the same steps for assembly and binning of respective metagenomic sequences. (TIF 112 kb)

Additional file 3: **Table S4.** Summary for all high-quality bins presented in Table 2, analysis of multiple copies of 16S rRNAs detected in MaxBin and CONCOCT derived bins as well as evaluation of misassemblies in gold standards. (XLSX 28 kb)

Additional file 4: **Table S3.** Sample statistics for quality control, number of reads selected and simulated for gold standards. (XLSX 25 kb)

Additional file 5: **Table S1.** Assembly Statistics of Megahit, MetaSPAdes and SPAdes for Sacramento, Boston samples and their respective in silico gold standards and pools. (XLSX 38 kb)

Additional file 6: **Table S2.** Bin Statistics of CheckM, barrnap (rRNAs) and tRNAscan-SE (tRNAs) for Sacramento, Boston samples and their respective in silico gold standards and pools. (XLSX 115 kb)

Additional file 7: **Figure S2.** Nonpareil Plot from Sacramento. A redundancy based estimation of average coverage for each sample from Sacramento is computed using Nonpareil [42]. Circles mark the actual sequencing effort of respective samples while the light red dotted line marks an estimated average coverage of 0.95. (TIFF 3164 kb)

Additional file 8: **Figure S3.** Nonpareil Plot from Boston grip, seats and back of seats. A redundancy based estimation of average coverage for selected samples from Boston is computed using Nonpareil [42]. Circles mark the actual sequencing effort of respective samples while the light red dotted line marks an estimated average coverage of 0.95. (TIFF 3164 kb)

Additional file 9: **Figure S4.** Nonpareil Plot from Boston poles and touchscreens. A redundancy based estimation of average coverage for selected samples from Boston is computed using Nonpareil [42]. Circles mark the actual sequencing effort of respective samples while the light red dotted line marks an estimated average coverage of 0.95. (TIFF 3164 kb)

Additional file 10: **Figure S5.** Nonpareil Plot from New York. A redundancy based estimation of average coverage for selected samples from New York is computed using Nonpareil [42]. Circles mark the actual sequencing effort of respective samples while the light red dotted line marks an estimated average coverage of 0.95. (TIFF 3164 kb)

Additional file 11: **Figure S6.** Nonpareil Plot from New York. A redundancy based estimation of average coverage for selected samples from New York is computed using Nonpareil [42]. Circles mark the actual sequencing effort of respective samples while the light red dotted line marks an estimated average coverage of 0.95. (TIFF 3164 kb)

Additional file 12: **Figure S7.** Assembly Statistics for in silico gold standards from Sacramento, Boston and New York. Assembly statistics for Megahit, MetaSPAdes and SPAdes from selected real and respective in silico gold standards (gs) for each surface type and cities are shown. Statistics are computed from all contigs above 500 bp in length. (TIFF 3164 kb)

## Abbreviations

AAI: Average Amino acid Identity
ANI: Average Nucleotide Identity
CAMDA: Critical Assessment of Massive Data Analysis
CAMI: Critical Assessment of Metagenome Interpretation
HMP: Human Microbiome Project
MIMAG: Minimum Information about a Metagenome-Assembled Genome
