# Supplementary figures and images for: Assessment of urban microbiome assemblies with the help of targeted in silico gold standards
Source: Biol Direct. 2018 Oct 12;13:22. doi: 10.1186/s13062-018-0225-6 (PMC6889603; doi:10.1186/s13062-018-0225-6)

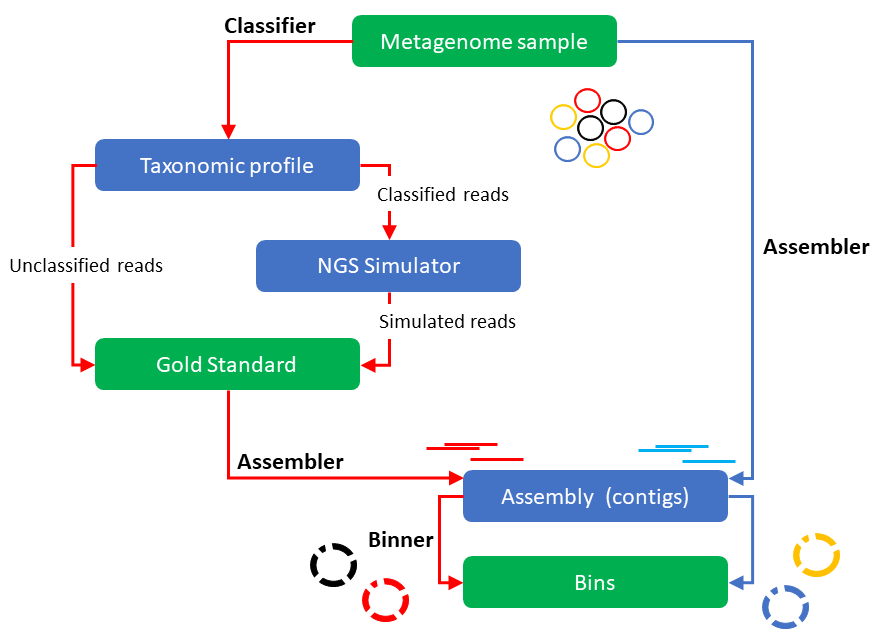

Supplement: Supplementary file 2 — Figure S1. Workflow for in silico gold standards. To create targeted in silico gold standards, all reads from a metagenome sample are classified. All reads classified to a taxonomic level or lower, i.e. bacterial, are simulated in silico in the exact same read counts from respective reference genomes. All simulated reads are then used to replace all classified bacterial reads, creating a gold standard maintaining composition of read errors, contamination and other characteristics of a specific sample, while providing known truth about a taxonomic domain of interest. All real samples together with their gold standard counterparts are processed in parallel using the same steps for assembly and binning of respective metagenomic sequences. (TIF 112 kb) [file 13062_2018_225_MOESM2_ESM.tif]

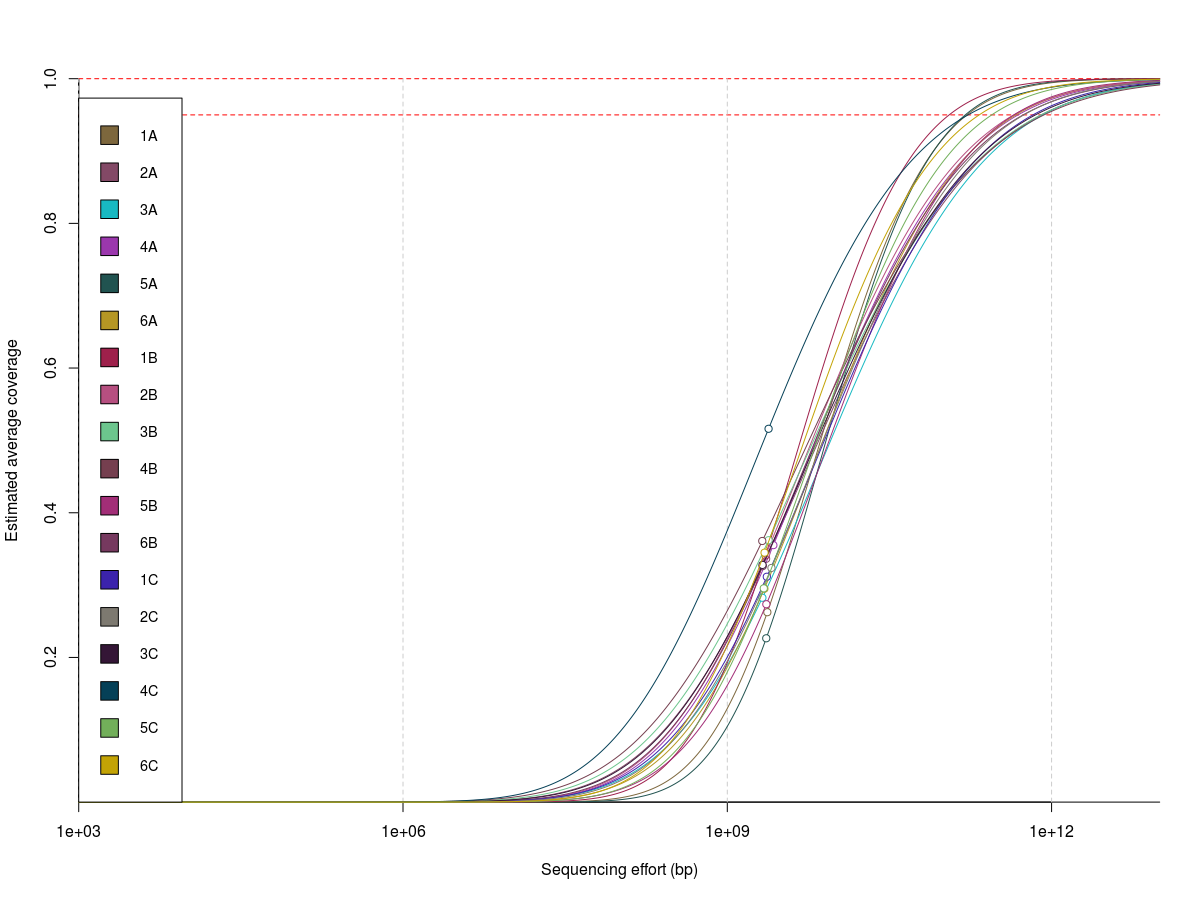

Supplement: Supplementary file 7 — Figure S2. Nonpareil Plot from Sacramento. A redundancy based estimation of average coverage for each sample from Sacramento is computed using Nonpareil [42]. Circles mark the actual sequencing effort of respective samples while the light red dotted line marks an estimated average coverage of 0.95. (TIFF 3164 kb) [file 13062_2018_225_MOESM7_ESM.tiff]

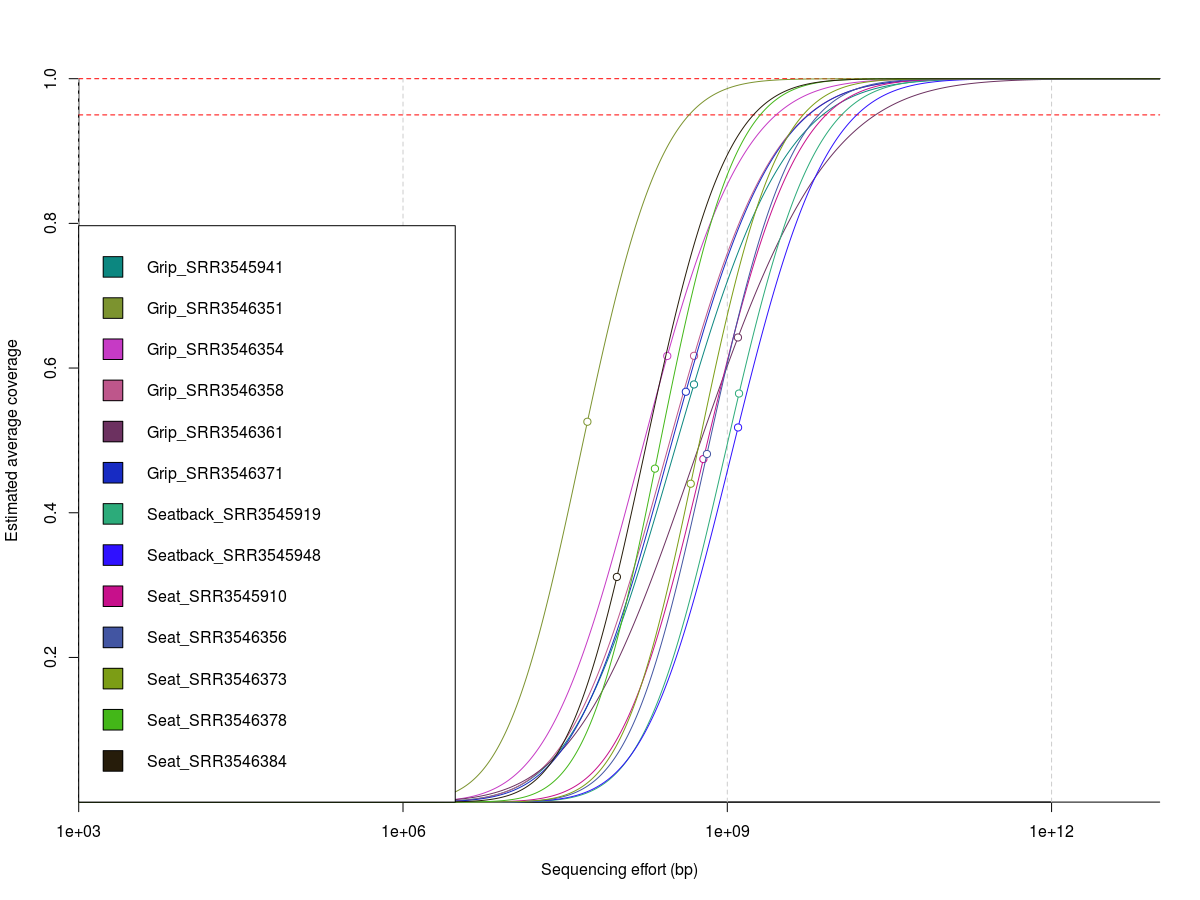

Supplement: Supplementary file 8 — Figure S3. Nonpareil Plot from Boston grip, seats and back of seats. A redundancy based estimation of average coverage for selected samples from Boston is computed using Nonpareil [42]. Circles mark the actual sequencing effort of respective samples while the light red dotted line marks an estimated average coverage of 0.95. (TIFF 3164 kb) [file 13062_2018_225_MOESM8_ESM.tiff]

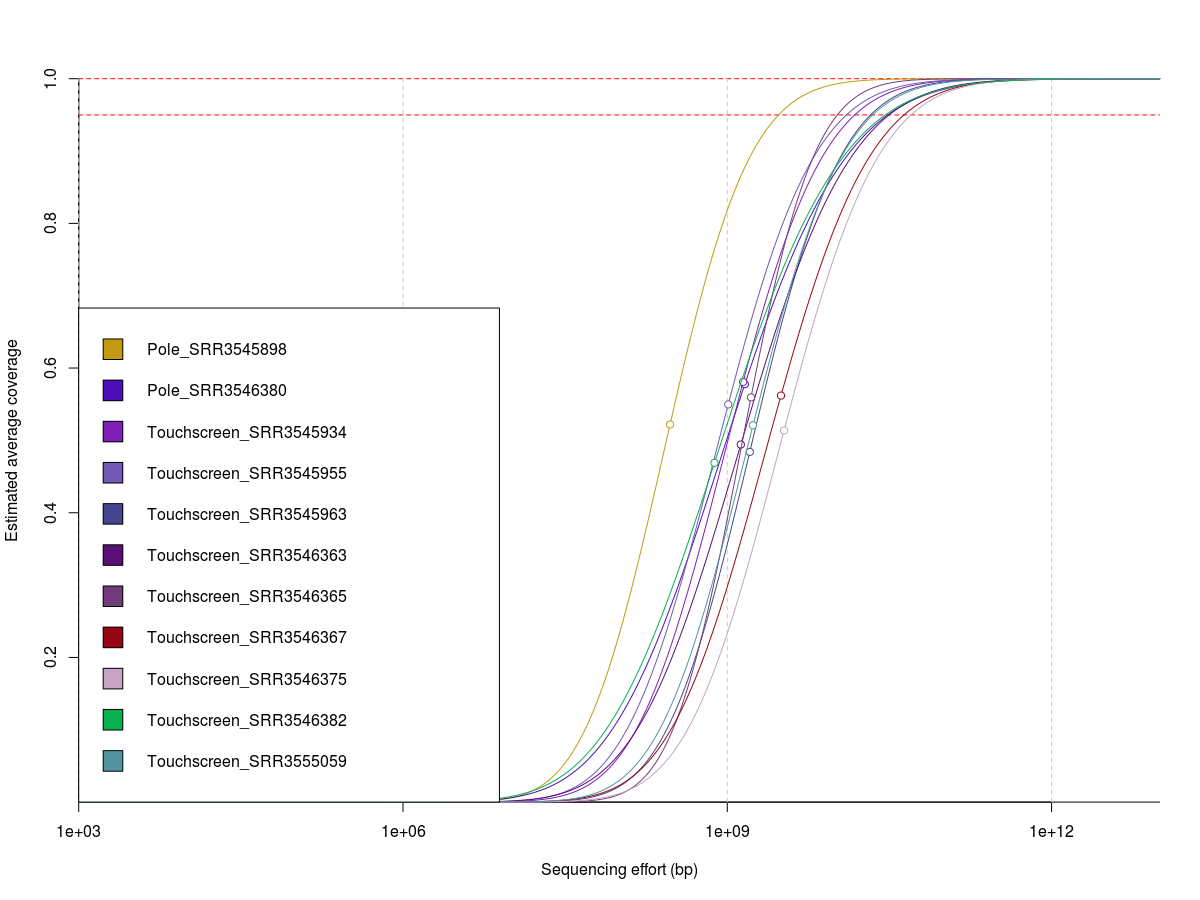

Supplement: Supplementary file 9 — Figure S4. Nonpareil Plot from Boston poles and touchscreens. A redundancy based estimation of average coverage for selected samples from Boston is computed using Nonpareil [42]. Circles mark the actual sequencing effort of respective samples while the light red dotted line marks an estimated average coverage of 0.95. (TIFF 3164 kb) [file 13062_2018_225_MOESM9_ESM.tiff]

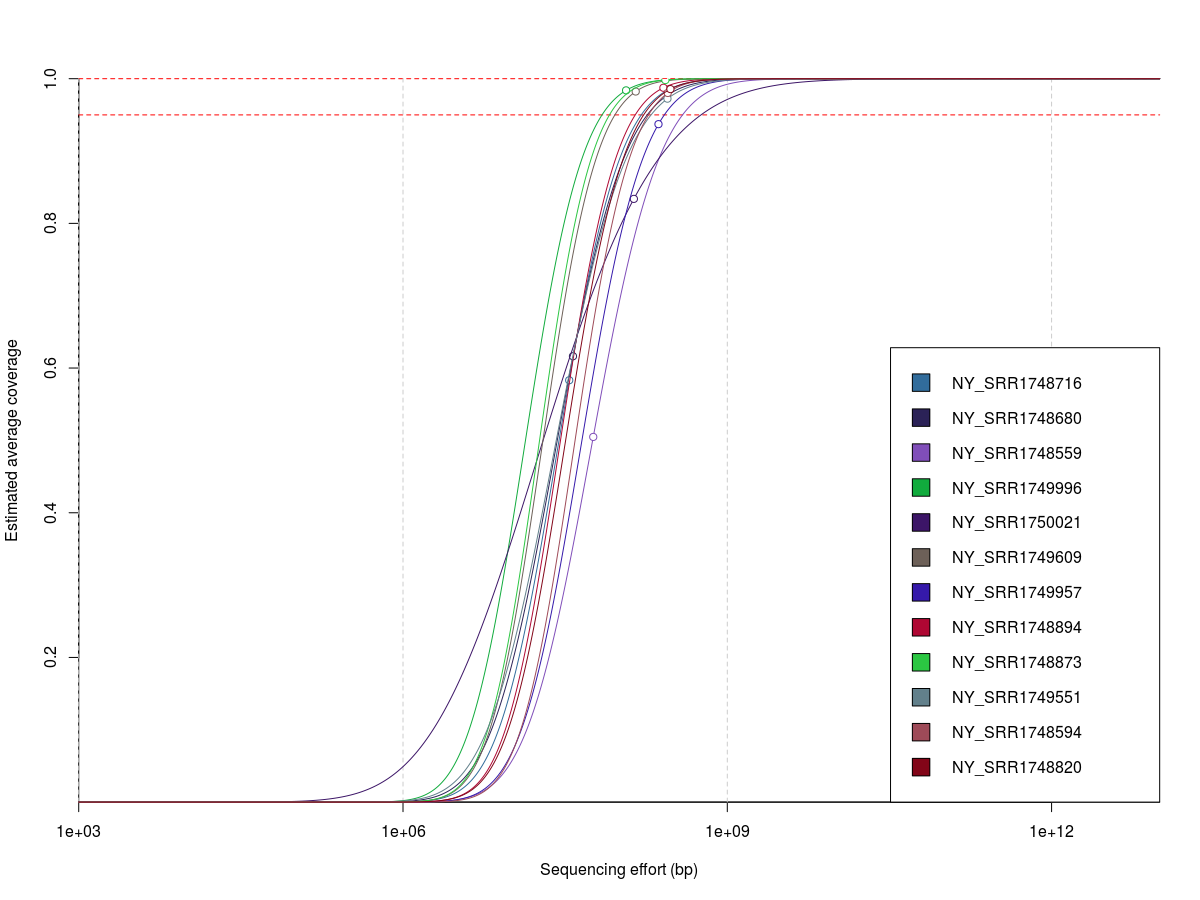

Supplement: Supplementary file 10 — Figure S5. Nonpareil Plot from New York. A redundancy based estimation of average coverage for selected samples from New York is computed using Nonpareil [42]. Circles mark the actual sequencing effort of respective samples while the light red dotted line marks an estimated average coverage of 0.95. (TIFF 3164 kb) [file 13062_2018_225_MOESM10_ESM.tiff]

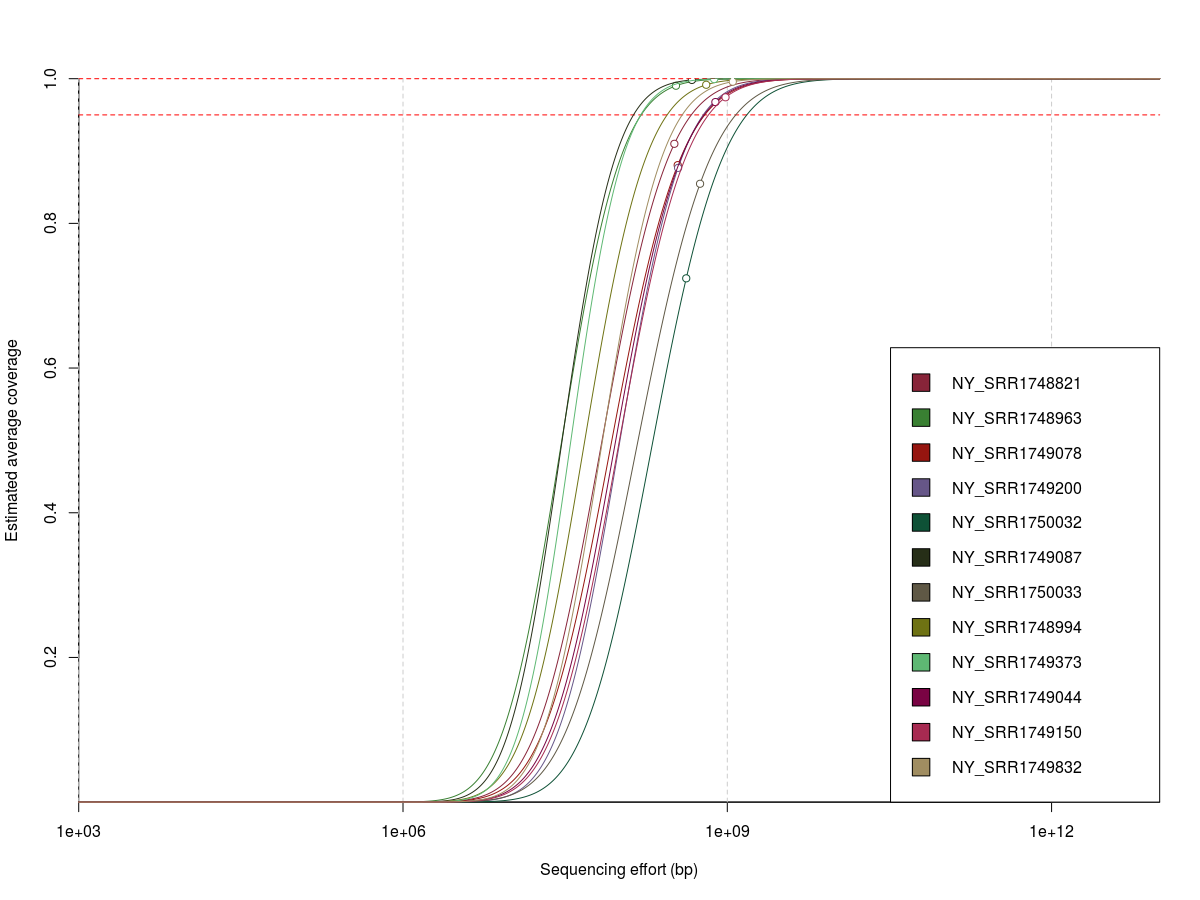

Supplement: Supplementary file 11 — Figure S6. Nonpareil Plot from New York. A redundancy based estimation of average coverage for selected samples from New York is computed using Nonpareil [42]. Circles mark the actual sequencing effort of respective samples while the light red dotted line marks an estimated average coverage of 0.95. (TIFF 3164 kb) [file 13062_2018_225_MOESM11_ESM.tiff]

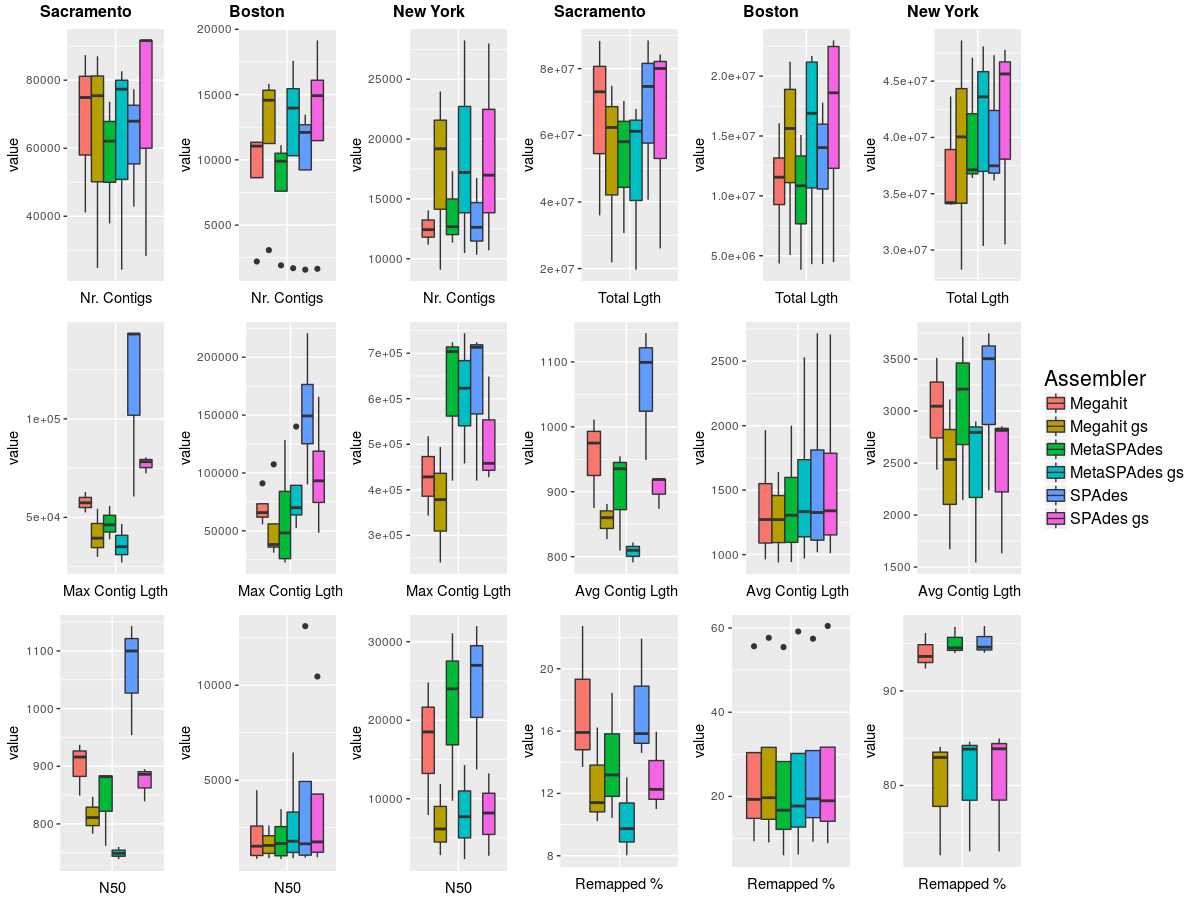

Supplement: Supplementary file 12 — Figure S7. Assembly Statistics for in silico gold standards from Sacramento, Boston and New York. Assembly statistics for Megahit, MetaSPAdes and SPAdes from selected real and respective in silico gold standards (gs) for each surface type and cities are shown. Statistics are computed from all contigs above 500 bp in length. (TIFF 3164 kb) [file 13062_2018_225_MOESM12_ESM.tiff]
